# EPHA3 regulates the multidrug resistance of small cell lung cancer via the PI3K/BMX/STAT3 signaling pathway

**DOI:** 10.1007/s13277-016-5048-4

**Published:** 2016-04-21

**Authors:** Juan Peng, Qiongyao Wang, Huanxin Liu, Minting Ye, Xiaoxia Wu, Linlang Guo

**Affiliations:** 1 Department of Pathology, The Third Affiliated Hospital of Guangzhou Medical University, Guangzhou, 510150 China; 2 Department of Pathology, Zhujiang Hospital of Southern Medical University, 253 Gongye Road, Guangzhou, 510282 China; 3 Department of Pathology, Armed Police Hospital of Guangdong Province, Guangzhou, 510507 China

**Keywords:** Small cell lung cancer (SCLC), Multidrug resistance (MDR), EPHA3, Apoptosis

## Abstract

Multidrug resistance (MDR) is a major obstacle to the treatment of small cell lung cancer (SCLC). EPHA3 has been revealed to be the most frequently mutated Eph receptor gene in lung cancer with abnormal expression. Growing evidence indicates that the signaling proteins of EPHA3 downstream, including PI3K, BMX and STAT3, play crucial roles in tumorigenesis and cancer progression. To explore the possible role of EPHA3 in MDR, we assessed the influence of EPHA3 on chemoresistance, cell cycle, apoptosis, and tumor growth, as well as the relationship between EPHA3 and the expression of PI3K, BMX, and STAT3 in SCLC. We observed that overexpression of EPHA3 in SCLC cells decreased chemoresistance by increasing apoptosis and inducing G0/G1 arrest, accompanied by reduced phosphorylation of PI3K/BMX/STAT3 signaling pathway. Knockdown of EPHA3 expression generated a resistant phenotype of SCLC, as a result of decreased apoptosis and induced G2/M phase arrest. And re-expression of EPHA3 in these cells reversed the resistant phenotype. Meanwhile, increased phosphorylation of PI3K/BMX/STAT3 signaling pathway was observed in these cells with EPHA3 deficiency. Notably, both PI3K inhibitor (LY294002) and BMX inhibitor (LFM-A13) impaired the chemoresistance enhanced by EPHA3 deficiency in SCLC cell lines. Furthermore, EPHA3 inhibited growth of SCLC cells in vivo and was correlated with longer overall survival of SCLC patients. Thus, we first provide the evidences that EPHA3 is involved in regulating the MDR of SCLC via PI3K/BMX/STAT3 signaling and may be a new therapeutic target in SCLC.

**Electronic supplementary material:**

The online version of this article (doi:10.1007/s13277-016-5048-4) contains supplementary material, which is available to authorized users.

## Introduction

Lung cancer is the leading cause of cancer-related death worldwide (World Health Organization. Cancer. 2012; http://globocan.iarc.fr). Among lung cancer types, small cell lung cancer (SCLC), the undifferentiated type, accounts for approximately 15 % of cases. The extreme aggressiveness and poor prognosis of SCLC are mainly due to its early and widespread metastases and development of multidrug resistance (MDR) to chemotherapy [1–4]. Mechanisms of MDR include decreased drug accumulation, drug inactivation, enhanced DNA repair, and abnormal cell apoptosis pathways. However, the molecular mechanisms involved in MDR processes of SCLC are not fully understood.

The Eph receptor family is the largest subgroup of the receptor tyrosine kinase (RTK) family. Eph receptors and their membrane-bound ligands, the ephrins, play critical roles in embryonic development, postnatal growth and pathogenesis [5–7]. The Eph receptor family is composed of two subclasses (A and B) and 16 members (A1-A10, B1-B6). EPHA3 (3p11.2) encodes a transmembrane protein with 983 amino acids that is widely expressed during embryonic development, overexpressed in the nervous system and heart, and exhibits lower expression in the brain, lung, bladder, prostate, and colon [8–13]. Studies have shown that EPHA3 exhibits abnormal expression in a variety of tumors, such as colorectal carcinoma, glioblastoma multiforme, melanoma, and hepatocellular carcinoma [14–17]. A recent collaborative study of 188 lung adenocarcinomas by Ding et al. revealed that EPHA3 is the Eph receptor found to be most frequently mutated in lung cancer, with 19 missense mutations identified so far and a mutation rate of 6 % [18]. Zhuang et al. further investigated the EPHA3 mutations and identified an EPHA3 mutation-associated gene signature in lung cancer that was associated with poor patient survival. Moreover, EPHA3 gene copy numbers and/or expression levels were decreased in tumors from large cohorts of patients with lung cancer. Re-expression of wild-type EPHA3 in human lung cancer lines increased apoptosis by suppression of AKT activation in vitro and inhibited the growth of tumor xenografts [19]. Although Ross et al. detected 98 SCLC samples by next-generation sequencing and found amplifications of EPHA3 genes in three cases of SCLC [20], EPHA3 associated with SCLC has rarely been published previously and little is known about the role of EPHA3 genes in chemoresistance.

RTKs, which are the major regulators of signal transduction pathways, are associated with cellular proliferation, apoptosis, and tumorigenesis. It is well documented that RTKs are involved in regulating the main functions of cells though the phosphatidylinositol-3 kinase (PI3K) signaling pathway [21, 22]. The PI3Ks are a family of intracellular lipid kinase enzymes, including class I, II, and III enzymes, which control a core cellular signaling and regulatory network and play a pivotal role in metabolism, growth, and survival in the cell. Class I PI3Ks are further divided into subclass IA (PI3Kα, PI3Kβ, and PI3Kδ isoforms) and IB (PI3Kγ isoform). PI3Kα is a heterodimer consisting of one regulatory subunit (p85α, encoded by PIK3R1) and one 110-kDa catalytic subunit (p110α, encoded by PIK3CA) that receives regulatory stimuli from transmembrane receptors via tyrosine kinase. In the p110α/p85α-PI3K complex, p85α is necessary for the cellular stability of PI3Kα and the nSH2 domain of p85 specifically interacts with the kinase domain of the EPH receptor [23–29].

Bone marrow kinase in chromosome X (BMX), a non-receptor tyrosine kinase member of the Tec kinase family, contains a PH-like domain that mediates membrane targeting by binding to phosphatidylinositol 3,4,5-triphosphate (PIP3) and an SH2 domain that binds to tyrosine-phosphorylated proteins and functions in signal transduction [30–32]. BMX was confirmed to be a critical downstream target of the constitutively active PI3-kinase in PTEN-deficient prostate cancer cells, and BMX was shown to activate the signal transducer and activator of transcription 3 (STAT3) signaling to maintain self-renewal and tumorigenic potential of glioblastoma stem cells [33, 34]. Activation of STAT3 was shown to be closely associated with cell apoptosis. STAT3 positively regulates cell survival by inducing Bcl-2 and Bcl-XL to repress apoptosis; inversely, STAT3 degradation and inhibition causes increased apoptosis [35–39]. Whether EPHA3 influenced cell apoptosis potentially through PI3K/BMX/STAT3 signaling to modulate MDR of SCLC is still unclear.

To better understand the biological function of EPHA3 in SCLC, we investigated its potential role in tumor growth and chemoresistance through loss- and gain-of-function approaches in human SCLC cell lines (H69, H69AR, H446, H146, and H1688). Moreover, we investigated the relationship between EPHA3 expression and the expression of PI3K/BMX/STAT3 signaling pathway. Finally, we detected EPHA3 expression in human SCLC tissues and evaluated the relevance of EPHA3 expression with clinical prognosis of SCLC patients. Therefore, we sought to identify the role of EPHA3 in SCLC progression and chemoresistance and its potential signaling pathway.

## Materials and methods

### Cell culture

The human SCLC cell line NCI-H69 and the drug-resistant sublines NCI-H69AR, NCI-H446, NCI-H146, and NCI-H1688 were purchased from the American Type Culture Collection (ATCC; Rockville, MD, USA) and maintained in RPMI 1640 medium (HyClone, Logan, UT, USA) with 10 % fetal calf serum (HyClone) in an incubator at 37 °C with 5 % CO_2_. The H69AR subline was maintained in a 5 μg/L final concentration of doxorubicin (Jiangshu, China) and transferred to drug-free media for at least 2 weeks before any experiment.

### Cell transfection

For stable transfections, which were selected for follow-up study, the EPHA3 expression plasmid EPHA3-PEX2-EcoRI/BamHI and the EPHA3-PEX2 empty plasmid (Genepharma, Shanghai, China), as well as the EPHA3-short hairpin RNA (shRNA) and the EPHA3 shRNA negative control (Genepharma), respectively, were transfected into NCI-H69, NCI-H69AR, NCI-H446, NCI-H146, and NCI-H1688 cells using Lipofectamine 2000 (Invitrogen, Carlsbad, CA, USA) and OPTI-MEM (Gibco, Gland Island, NY, USA).

### RNA isolation and quantitative reverse transcription-PCR

Total RNA was isolated from cell lines using RNAiso Plus (Takara, Dalian, China) according to the manufacturer’s instructions and the concentration of RNA was determined using a NanoDrop 2000 spectrophotometer (Thermo Scientific, Rockford, IL, USA). cDNA synthesis was carried out according to the PrimeScript RT reagent Kit with gDNA Eraser (Takara). qRT-PCR for EPHA3 was performed in triplicate with SYBR Premix Ex Taq^TM^ II (Takara) on an Illumina-Eco Real-Time PCR System (illumina, San Diego, CA, USA) using the following primers: human EPHA3 forward, 5′-GTTCTCTGGGAGGTGATGTCTT-3′ and reverse, 5′-GGGTCTGTTGTTCCTGTCTTTC-3′ (Sangon Biotech, Inc., Shanghai, China); GAPDH (endogenous control) forward, 5′-AGAAGGCTGGGGCTCATTTG-3′ and reverse, 5′-AGGGGCCATCCACAGTCTTC-3′ (Sangon Biotech, Inc.).

### Western blotting analysis

Total protein was isolated with a total protein extraction kit (Keygene, Nanjing, China) and quantitated using a BCA assay kit (Keygene). The protein lysates were separated using 8 or 10 % SDS-PAGE gels and electrophoretically transferred to polyvinylidene difluoride (PVDF) membranes. After antigen blocking, the transferred PVDF membrane was incubated with primary antibodies for EPHA3 (clone 3A12, 3.5 μg/mL, Abnova, Taiwan), phospho-PIK3R1 (pTyr467, 1:500, Sigma-Aldrich, St. Louis, MO, USA), total PI3K p85α (clone Ab6, 1 μg/mL, Abcam, Cambridge, England), phospho-BMX (Tyr40, 1:1000, Cell Signaling technology), total BMX (Y396, 1:1000, Abcam, Cambridge, England), phospho-Stat3 (Tyr705, D3A7, 1:1000, Cell Signaling technology), total STAT3 (clone 4D6, 10 μg/mL, Abnova), and GAPDH (internal control, 1D4, 1:1000, EarthOx, San Francisco, CA, USA) overnight at 4 °C with gentle shaking. The secondary goat anti-Rabbit-IgG (EarthOx) or goat anti-Mouse-IgG (EarthOx) HRP AffiniPure antibody was added correspondingly at a 1:2000 dilution. After chemiluminescence, the intensity of the protein fragments was quantified using Quantity One software (4.5.0 basic, Bio-Rad Laboratories, Hercules, CA, USA).

### Cell counting kit-8 (CCK-8) assay

Cells were reseeded in 96-well plates at 5 × 10^3^ cells per well for NCI-H69, NCI-H69AR, NCI-H446, and NCI-H1688 and 20 × 10^3^ cells per well for NCI-H146. After adherence of stably transfected cells, cells were treated with drugs for 24 h. A total of three chemotherapy drugs [Adriamycin (ADM; Jiangshu, China), Cisplatin (DDP; Shandong, China), and Etoposide (VP-16; Jiangshu, China)] were used with different drug concentration gradients. The absorbance at 450 nm was measured after incubation with 10 μL CCK-8 reagent (Dojindo, Kumamato, Japan) for 4 h. The cells incubated without drugs were set at 100 % survival and were used to calculate the IC50 (ug/mL) concentration for each chemotherapeutic drug. The assay was conducted in five replicate wells for each sample and three independent experiments were performed.

### Flow cytometric analysis

Cells were treated with ADM (4.6 μM), DDP (16.7 μM), and VP-16 (34 μM) [40] for 24 h and then kept in drug-free media for 6 h. Cells were collected for the early apoptosis assay performed using an Annexin V/propidium iodide detection kit (Becton-Dickinson (BD), San Jose, CA, USA) on a FACSCalibur system (BD). For the cell-cycle assay, the cells were collected and fixed in 70 % ethanol at 4 °C for 16 h and then stained with propidium iodide. Cellquest Pro software was used for apoptosis analysis and ModFit LT software was used for the analysis of the cell cycle. Cells in the quadrant of Annexin V^−^PI^−^ (lower left) represent viable cells, cells in the quadrant of Annexin V^−^PI^+^ (upper left) represent necrotic cells, cells in the quadrant of Annexin V^+^PI^+^ (upper right) represent late apoptotic and dead cells, and cells in the quadrant of Annexin V^+^PI^−^ (lower right) represent early apoptotic cells. Generally, apoptosis analysis is based mainly on the percentage of Annexin V^+^PI^−^ cells. All assays were carried out independently in triplicate.

### Clinical samples and immunohistochemistry

Sixty-one SCLC tumor tissues and 25 normal lung tissues were obtained from the Armed Police Hospital of Guangdong Province and the Third Affiliated Hospital of Guangzhou Medical University. All samples were confirmed as SCLC by pathologic examination, and tumor staging was further distinguished as limited disease (20 cases) versus extensive disease (41 cases) according to the Veterans Administration Lung Cancer Study Group system. Immunohistochemistry was performed under antigen retrieval conditions in 2-mM citric acid, 10-mM sodium citrate buffer, and at pH 6.0. Endogenous peroxidase was blocked by 3 % H_2_O_2_ for 30 min, and the sections were stained with rabbit polyclonal antibody against human EPHA3 (1: 400; Santa Cruz Biotechnology, Santa Cruz, CA, USA). Negative controls were performed by replacing the primary antibodies stated above with PBS. Stained sections were analyzed independently by two different pathologists. Positively stained EPHA3 was primarily located in the cytoplasm of cells and appeared as light brown and brown particles. The intensity of staining was scored manually (high, 3; medium, 2; low, 1; no staining, 0). The extent of staining was scored as 0 (0–10 %), 1 (11–25 %), 2 (26–50 %), or 3 (51–100 %), according to the percentages of the positive stained area in relation to the entire carcinoma-involved area or the entire normal sample area. The sum of the intensity and extent scores was used as the final staining score (0–6). Optimal cut off values were identified; a final staining score ≤1 indicated negative expression (−) and a final staining score ≥1 indicated positive expression (+).

### In vivo tumor growth

Male BALB/c nude mice aged 30–40 days (purchased from the Medical Experimental Animal Center of Guangdong Province, China) were raised under pathogen-free conditions. All procedures were performed according to the guidelines of the Association for Assessment and Accreditation of Laboratory Animal Care International. Cells in RPMI-1640 medium were subcutaneously inoculated into the shoulder-back of nude mice in the light of 1 × 10^7^/0.1 mL/site to establish the tumor model. The tumor volume was determined every 3 days over the course of 21 days by direct measurement with a sliding caliper and calculated by the equation (*V* = 0.4 × ab^2^, where *a* is the widest diameter of the tumor and *b* is the diameter perpendicular to *a*) to construct the growth curves of the tumors. Twenty-one days later, the mice were sacrificed and tumors were excised. Half of the tumor tissues were fixed with neutral phosphate-buffered formalin, followed by immunohistochemical examination. The other half of the tumors were washed with pre-chilled PBS, and the total protein of the tumors was isolated for subsequent Western blotting examination of p-STAT3 and total STAT3 protein.

### Statistical analysis

All data are shown as the mean ± standard deviation and were generated using SPSS 19.0 statistical software. One-way ANOVA analyses, *χ* ^2^ test, Kaplan-Meier survival analyses, and Cox regression analyses were used. *P* < 0.05 was considered significant.

## Results

### Overexpression of EPHA3 reinforces a sensitive phenotype

**Fig. 1:**
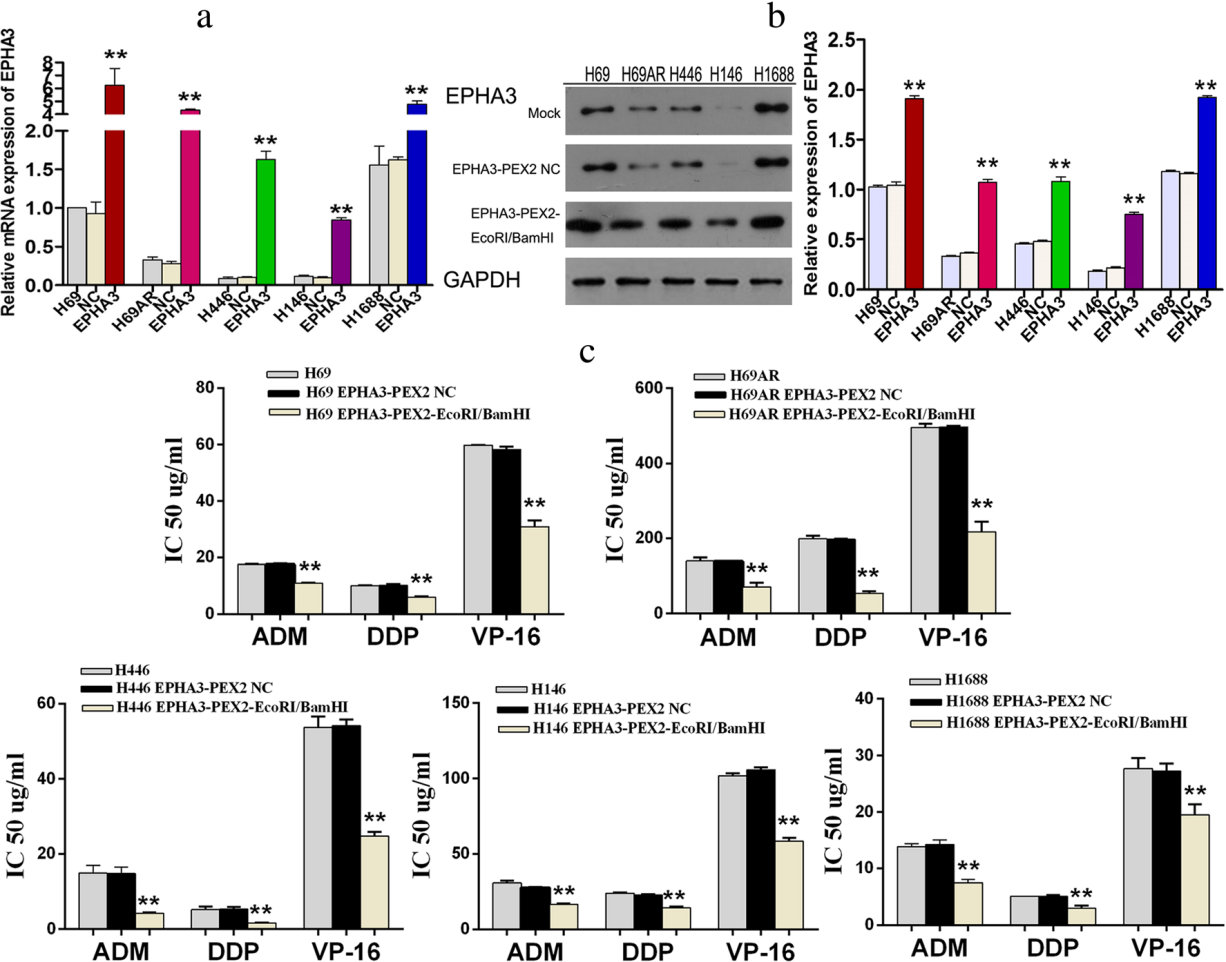
Overexpression of EPHA3 reinforced the chemosensitivity in SCLC cell lines. The differential expression of EPHA3 was assessed in H69, H69AR, H446, H146, and H1688 cells by qRT-PCR (**a**) and Western blotting (**b**). The higher expression of EPHA3 was detected in H69, H69AR, H446, H146, and H1688 cells transfected with plasmid EPHA3-PEX2-EcoRI/BamHI by qRT-PCR (**a**) and Western blotting (**b**) compared with mock and NC. The drug sensitivity of SCLC cell lines displayed as the IC50 values were measured in these SCLC cell lines after treatment with ADM, DDP, or VP-16 using the CCK-8 assay. Overexpression of EPHA3 in these SCLC cell lines led to a significant reduction in the IC50 values (**c**)

**Fig. 2:**
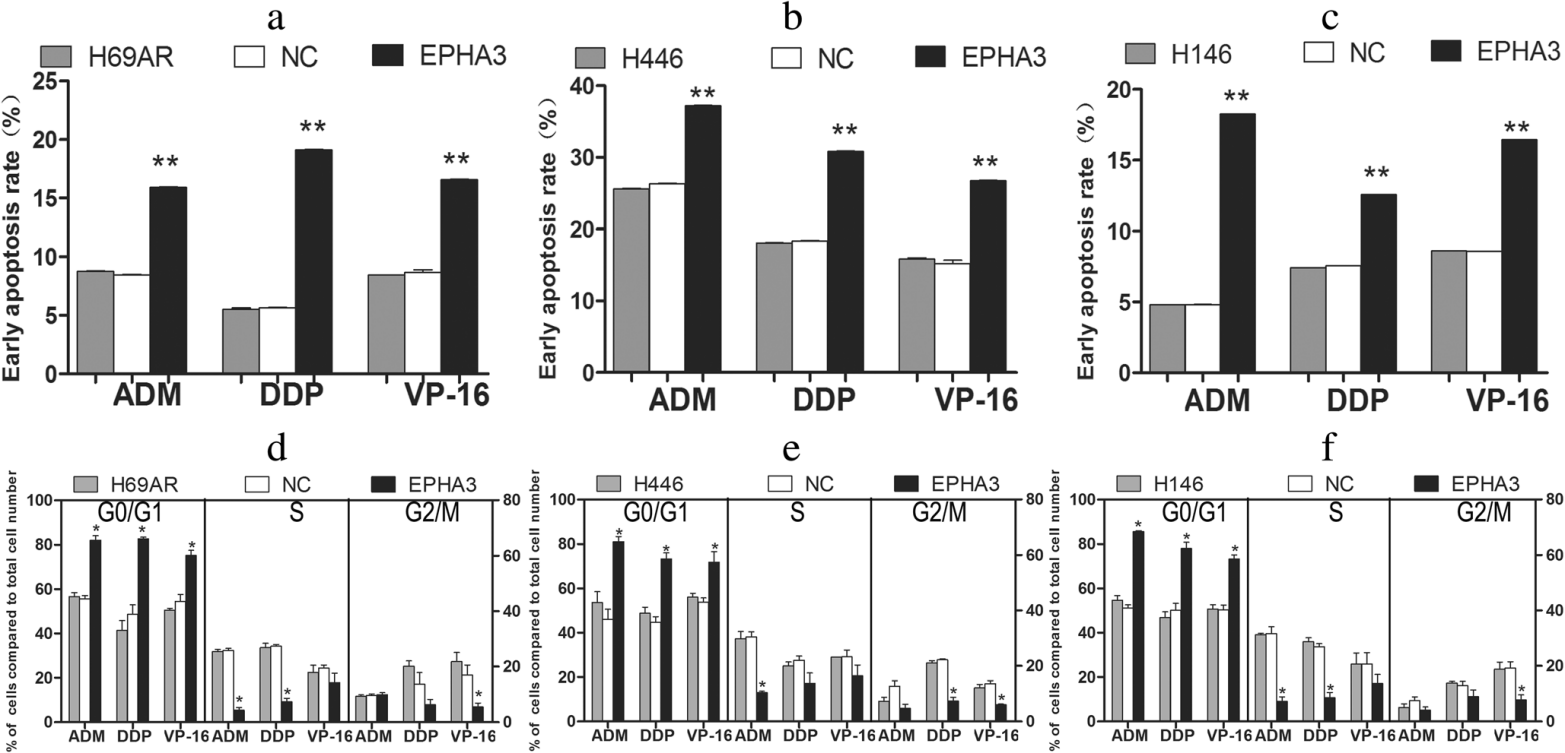
Overexpression of EPHA3 induced the cell early apoptosis rate and G0/G1 phase arrest. H69AR (**a**, **d**), H446 (**b**, **e**), and H146 (**c**, **f**) cells transfected with plasmid EPHA3-PEX2-EcoRI/BamHI or NC were detected by flow cytometric analysis after treatment with ADM, DDP, or VP-16

To investigate the effect of EPHA3 on SCLC chemoresistance, the expression of EPHA3 was initially detected in SCLC drug-resistant cells (H69AR) and drug-sensitive cells (H69, H446, H146, and H1688) by qRT-PCR and Western blotting. We found that EPHA3 expression at both the mRNA and protein level was significantly higher in H69 and H1688 cells than in H69AR, H446, and H146 cells (*P* < 0.05; Fig. 1a, b). We then transfected all 5 SCLC cell lines with the plasmid EPHA3-PEX2-EcoRI/BamHI, and the overexpression of EPHA3 in these cells was confirmed by qRT-PCR and Western blotting (**P < 0.05; Fig. 1a, b). CCK-8 assay revealed a significant reduction of IC50 values and increased sensitivity to ADM, DDP, or VP-16 in these cells transfected with EPHA3-PEX2-EcoRI/BamHI compared with relevant NC and mock cells (**P < 0.05; Fig. 1c). In addition, a significant induction of cell early apoptosis and G0/G1 cell-cycle arrest was observed in H69AR (*P* < 0.05; Fig. 2a, d), H446 (*P* < 0.05; Fig. 2b, e), and H146 cells with EPHA3 overexpression (*P* < 0.05; Fig. 2c, f; Supplementary Figure S1) by flow cytometry analysis. These findings suggested that EPHA3 sensitizes SCLC cells to chemotherapy drugs, and possibly plays critical roles in chemoresistance of SCLC cells.

### Knockdown of EPHA3 results in a resistant phenotype

**Fig. 3:**
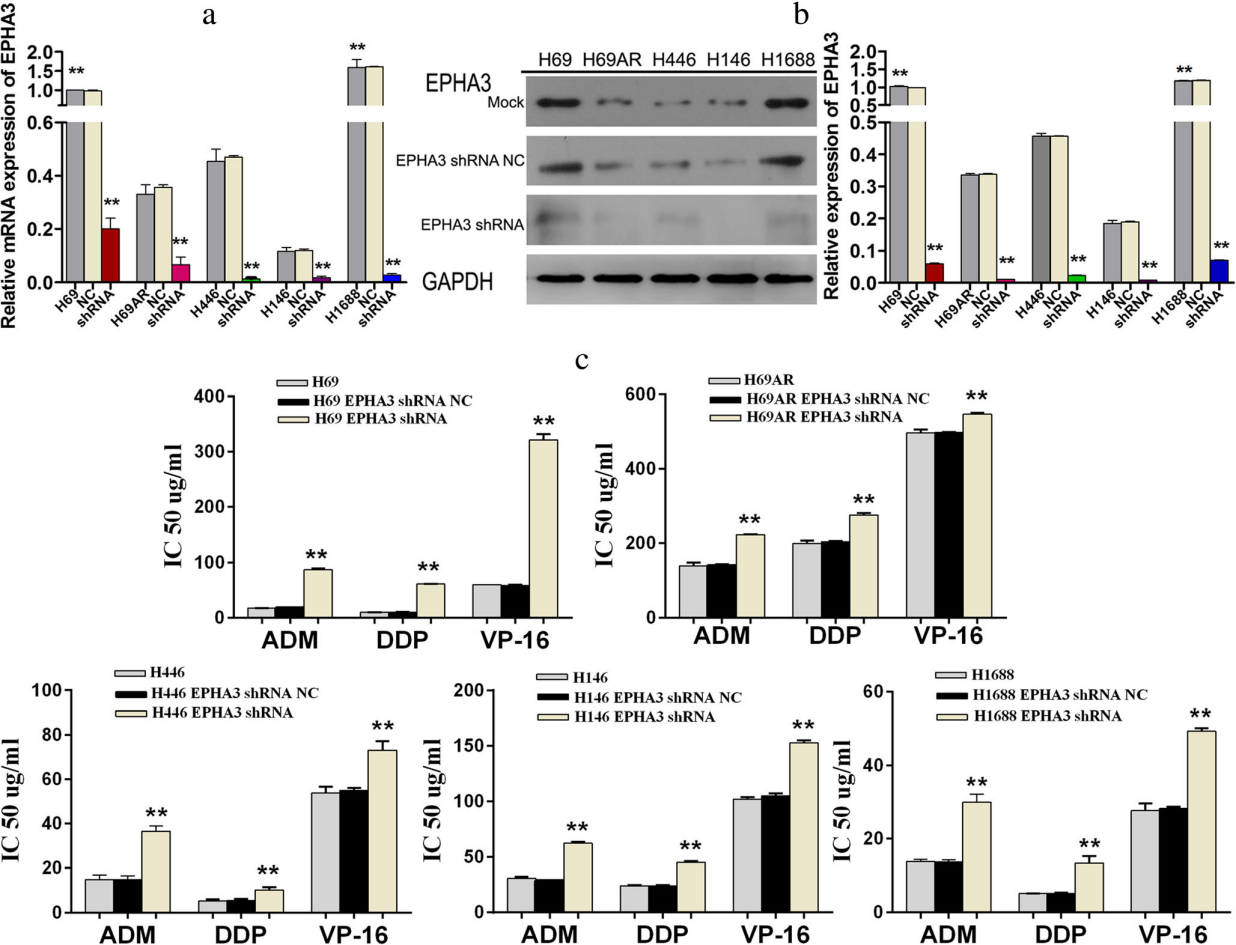
Knockdown of EPHA3 enhanced chemoresistance of SCLC cell lines. A significant EPHA3 deficiency was detected in these SCLC cell lines transfected with EPHA3 shRNA by qRT-PCR (**a**) and Western blotting (**b**). CCK-8 assay showed that knockdown of EPHA3 in these SCLC cell lines significantly increased the IC50 values (**c**)

**Fig. 4:**
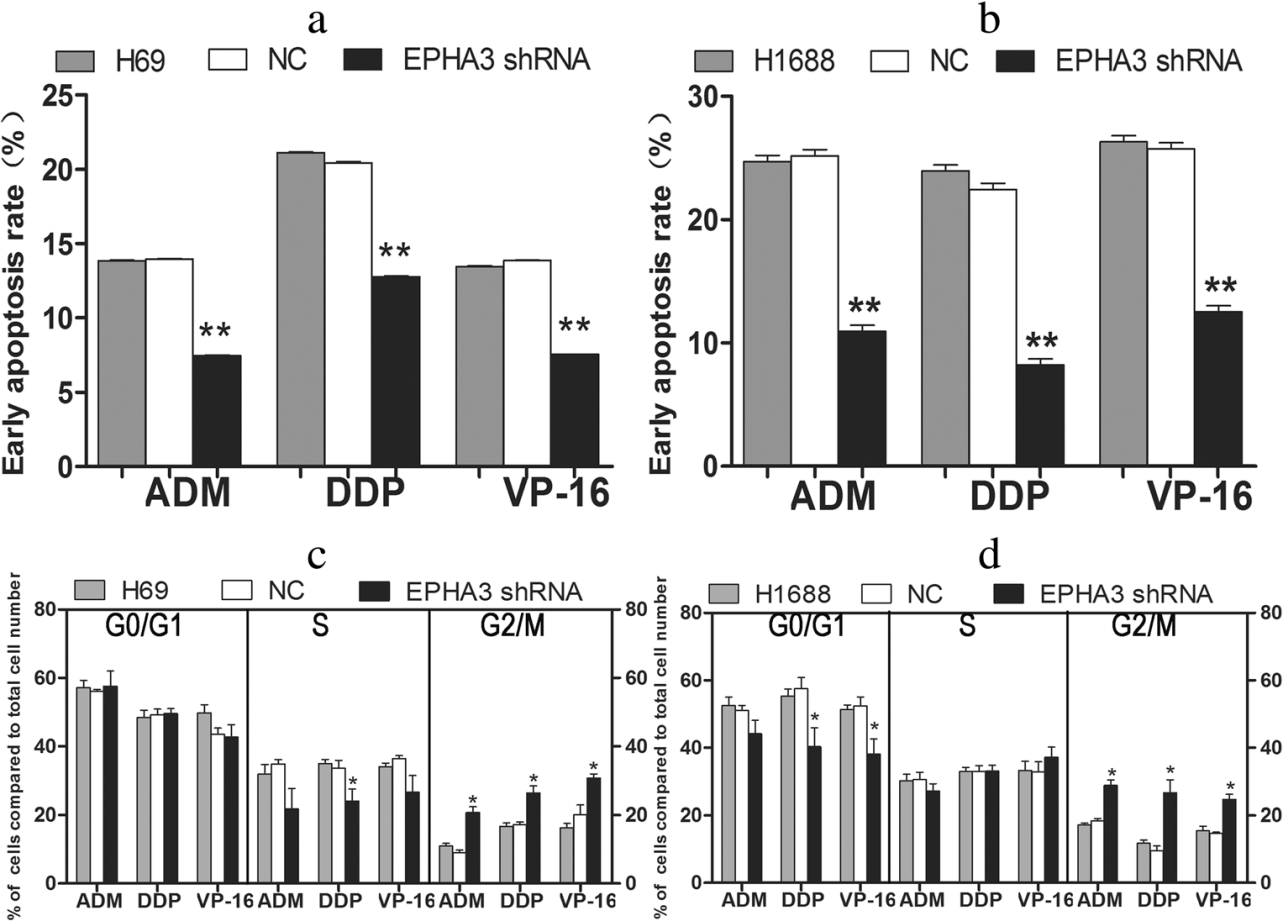
Knockdown of EPHA3 resulted in a reduced early apoptosis rate and G2/M cell-cycle arrest. Cell apoptosis and cell cycle were assayed by flow cytometric analysis after H69 (**a**, **c**) and H1688 (**b**, **d**) cells with EPHA3 deficiency were treated with ADM, DDP, or VP-16

To further verify the effect of EPHA3 on SCLC chemoresistance, we subsequently transfected SCLC cells with EPHA3 shRNAs (EPHA3-1690, −1286, −2635, −2934) and NC to knockdown the expression of EPHA3, and shRNA-1690 for H69 and H69AR cells, as well as −2934 for H446, H146, and H1688 cells (Supplementary Figure S2). The cells which showed the most significant inhibition were selected for follow-up study. All 5 SCLC cell lines transfected with EPHA3 shRNAs showed decreased expression of EPHA3 both at the mRNA and protein levels compared with relevant NC and mock cells (**P < 0.05; Fig. 3a, b). The IC50 values of drug-sensitive SCLC cells (H69, H446, H146, and H1688 cells) treated with chemotherapeutic drugs were significantly increased with the EPHA3 deficiency, while the drug-resistance of H69AR cells was further enhanced (**P < 0.05; Fig. 3c). Furthermore, we found that EPHA3 deficiency led to decreased cell apoptosis and induced G2/M cell-cycle arrest in H69 (*P* < 0.05; Fig. 4a, c) and H1688 cells (*P* < 0.05; Fig. 4b, d; Supplementary Figure S3) treated with the chemotherapeutic drugs stated above. As expected, the knockdown of EPHA3 results in the formation of a resistant phenotype in SCLC cell lines.

**Fig. 5:**
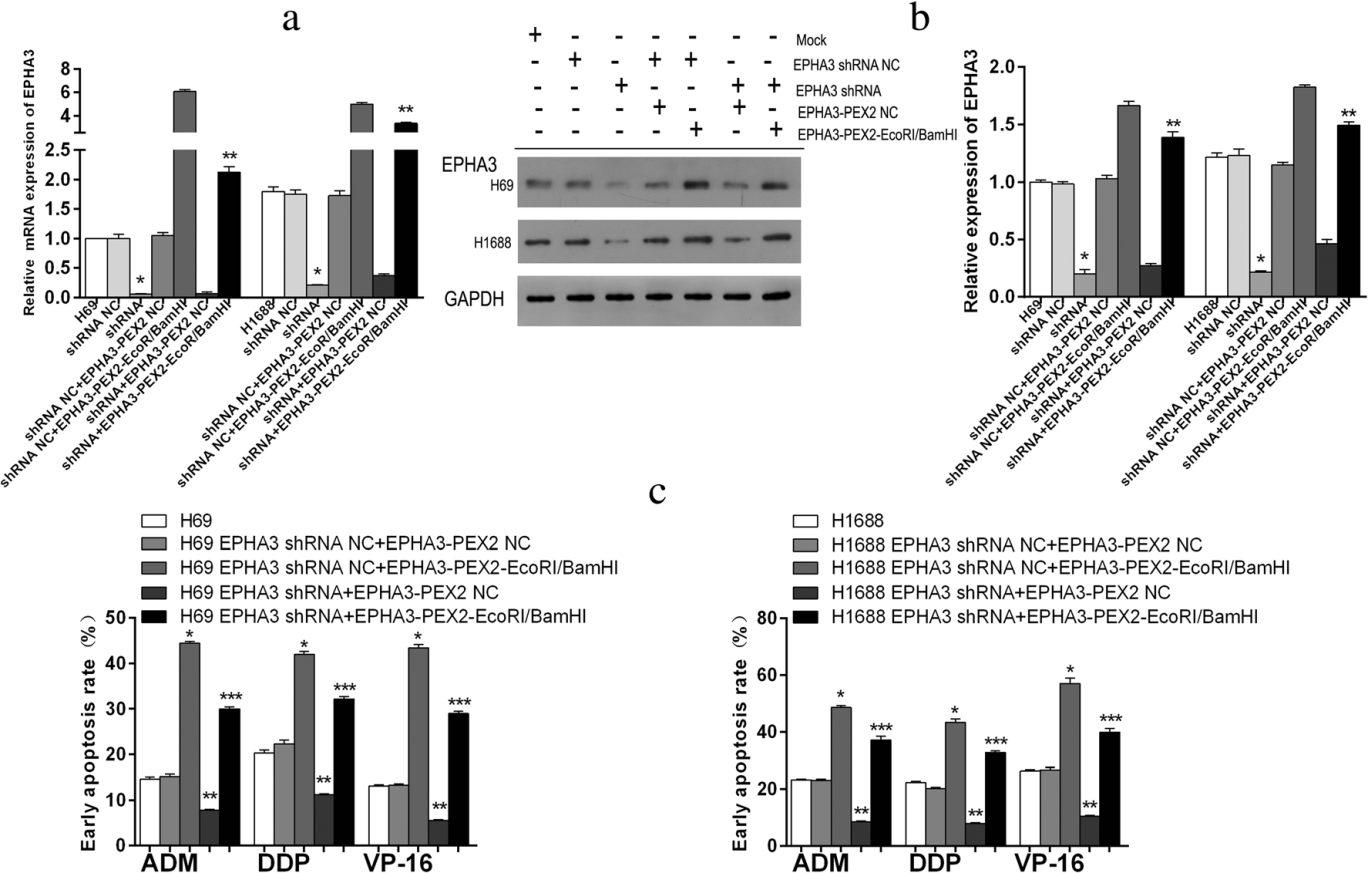
Re-expression of EPHA3 increased the cell early apoptosis rate in SCLC cell lines. After co-transfection, the expression of EPHA3 in H69 and H1688 cells with EPHA3 deficiency was detected to be re-expressed at both the mRNA and protein level by qRT-PCR (**a**) and Western blotting (**b**). In addition, after co-transfection, the cell early apoptosis rate of these EPHA3 deficiency cells was detected to be increased by flow cytometric analysis following treatment with ADM, DDP, or VP-16, in comparison with mock and NC cells, respectively (**c**)

To further determine whether EPHA3 indeed exerts its function on SCLC chemoresistance, we conducted the rescue experiment by co-transfecting the stably silenced cells (H69 EPHA3 shRNA and H1688 EPHA3 shRNA) and their corresponding EPHA3 shRNA NC cells with the plasmids EPHA3-PEX2-EcoRI/BamHI and EPHA3-PEX2 NC, respectively. As shown in Fig. 5, the downregulated expression of EPHA3 in H69 and H1688 cells was re-expressed at both the mRNA and protein level after co-transfection by qRT-PCR (**P < 0.05; Fig. 5a) and Western blotting (**P < 0.05; Fig. 5b). Flow cytometric analysis was conducted to test for cell apoptosis induced by chemotherapeutic drugs after co-transfection. In contrast to the reduction of cell apoptosis rate in H69 and H1688 cells caused by EPHA3 deficiency, the rate of cell apoptosis increased after co-transfection compared with mock cells and NC cells (***P < 0.05; Fig. 5c; Supplementary Figure S4), respectively. Collectively, these results suggested that EPHA3 has a reliable influence on the regulation of MDR in SCLC cell lines through the cell apoptosis pathway.

### EPHA3 modulates the protein expression of the PI3K/BMX/STAT3 signaling pathway

**Fig. 6:**
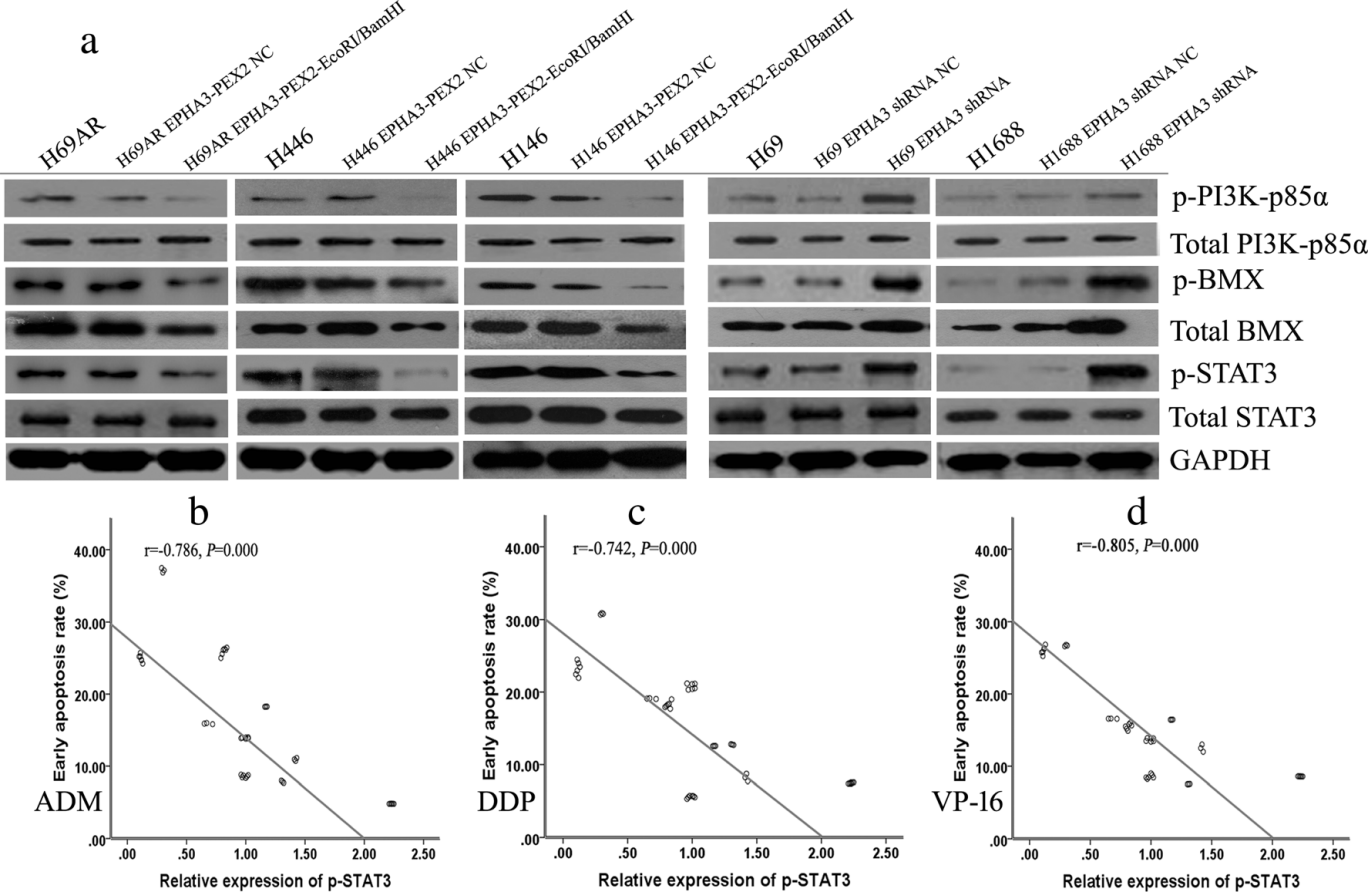
The protein expression of PI3K/BMX/STAT3 was modulated by the manipulation of EPHA3 expression in SCLC cell lines. Overexpression of EPHA3 in H69AR, H446, and H146 cells resulted in the decreased the expression of p-PI3K-p85α, p-BMX, total BMX, and p-STAT3 by Western blotting. By contrast, knockdown of EPHA3 in H69 and H1688 cells increased expression of p-PI3K-p85α, p-BMX, total BMX, and p-STAT3 protein levels (**a**). However, there was no significant difference in total PI3K-p85α and total STAT3 expression between the treatment groups. Furthermore, the expression level of p-STAT3 was negatively correlated with the cell early apoptosis rate induced by ADM (**b**), DDP (**c**), or VP-16 (**d**) in SCLC cell lines

The above observations prompted us to investigate the potential downstream signaling pathway of EPHA3 in chemoresistance of SCLC. To explore the modulation mechanisms of EPHA3 on MDR through the cell apoptosis pathway, we performed Western blotting analyses to further investigate whether EPHA3 was potentially involved in signaling through the PI3K/BMX/STAT3 pathway. The results showed that overexpression of EPHA3 in H69AR, H446, and H146 cells downregulated the expression of p-PI3K-p85α, p-BMX, total BMX, and p-STAT3 (*P* < 0.05), whereas knockdown of EPHA3 in H69 and H1688 cells increased the expression of p-PI3K-p85α, p-BMX, total BMX, and p-STAT3 protein levels (*P* < 0.05; Fig. 6a; Supplementary Figure S5). However, there was no significant difference in total PI3K-p85α and total STAT3 expression. These findings demonstrated that EPHA3 significantly reduces the phosphorylation of PI3K/BMX/STAT3 in SCLC cell lines. Interestingly, the expression level of p-STAT3 was observed to be negatively correlated with the cell early apoptosis rate induced by the three chemotherapeutic drugs in the SCLC cell lines (2-tailed Pearson correlation, *r* = −0.786, −0.742, −0.805; *P* < 0.05; Fig. 6b–d), which was consistent with the conclusions stated above.

**Fig. 7:**
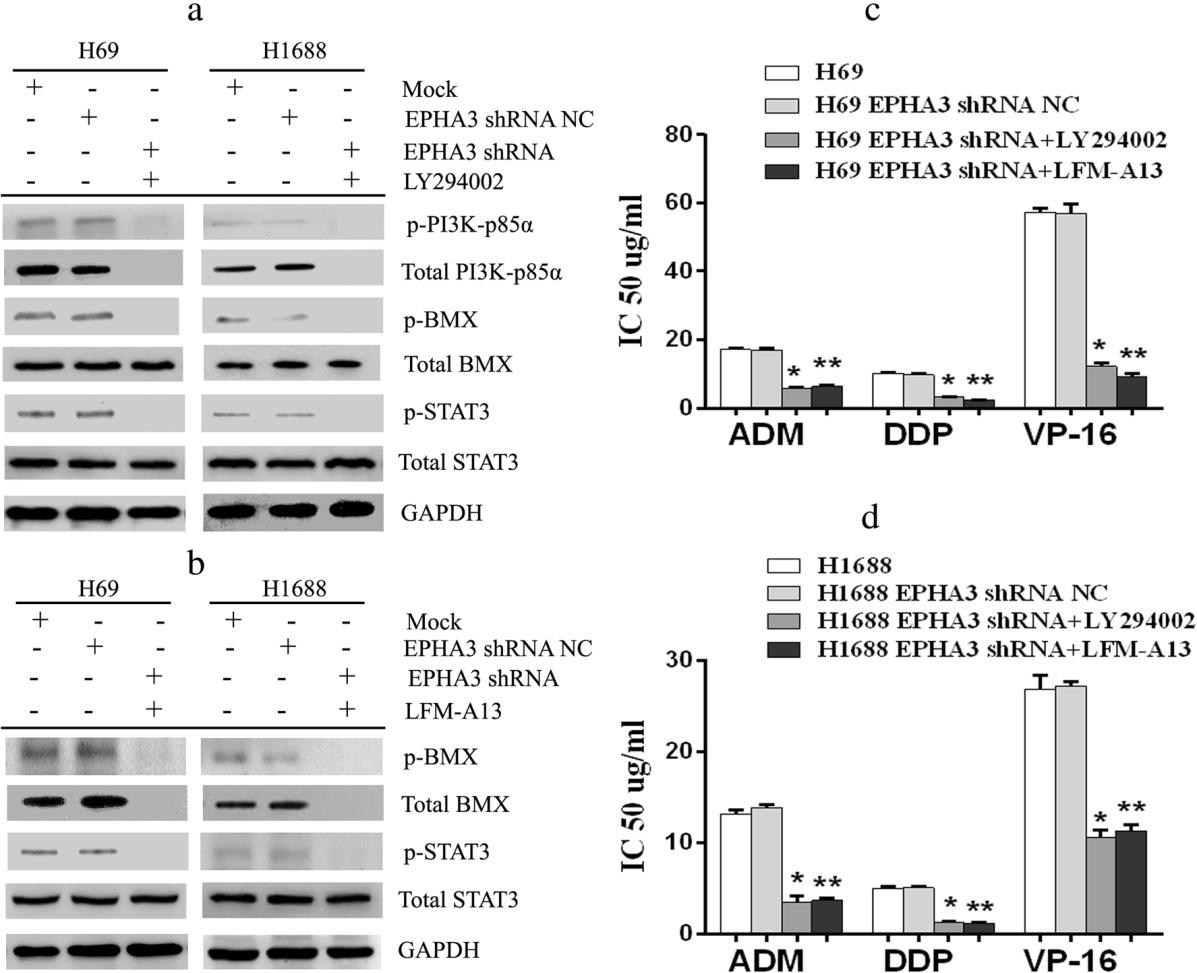
The signaling pathway inhibitors blocked the effect of EPHA3 deficiency on SCLC chemoresistance. After the stably silenced cells (H69 and H1688 EPHA3 shRNA) were treated with the PI3K-specific inhibitor LY294002 (10 μM, Sigma-Aldrich) for 2 h, we detected the expression of PI3K/BMX/STAT3 protein by Western blotting. The increased expression of p-BMX and p-STAT3 protein levels induced by EPHA3 shRNA in H69 and H1688 cells was blocked by the inhibition of PI3K/BMX pathway with LY294002 (**a**). The expression of p-PI3K-p85α and total PI3K-p85α in the stably silenced cells simply vanished under exposure to LY294002. Moreover, we evaluated the expression of BMX/STAT3 protein in these stably silenced cells by Western blotting following treatment with BMX inhibitor LFM-A13 (3 μM, Sigma-Aldrich) for 2 h. In the same way, the increased expression of p-STAT3 protein levels induced by EPHA3 shRNA in H69 and H1688 cells was blocked by LFM-A13 (**b**), accompanied by the disappearance of p-BMX and total BMX expression. Even more, the IC50 values of the stably silenced cells exposure to chemotherapeutic drugs were significantly decreased by CCK-8 assay, whether treated with LY294002 or LFM-A13 (**c**, **d**)

To further determine whether EPHA3 indeed regulates the expression of PI3K/BMX/STAT3 signaling pathway, the PI3K-specific inhibitor LY294002 was used to block PI3K/BMX pathway. The results showed that the increased expression of p-BMX and p-STAT3 protein levels induced by EPHA3 shRNA were reduced, even vanished significantly both in H69 and H1688 cells (*P* < 0.05; Fig. 7a; Supplementary Figure S6). The expression of p-PI3K-p85α and total PI3K-p85α in the stably silenced cells simply vanished under exposure to LY294002. We therefore proceeded to block BMX/STAT3 pathway with the BMX inhibitor LFM-A13. Similarly, LFM-A13 reversed the overexpression of p-STAT3 caused by EPHA3 shRNA in H69 and H1688 cells (*P* < 0.05; Fig. 7b; Supplementary Figure S6) and led to the disappearance of p-BMX and total BMX expression in these cells. We subsequently investigated the sensitivity of these stably silenced cells treated with the inhibitors in response to the three chemotherapy drugs by CCK-8 assay. The IC50 values of the stably silenced cells exposure to chemotherapeutic drugs were significantly decreased with the inhibition of PI3K/BMX or BMX/STAT3 pathway (*P* < 0.05; Fig. 7c, d). Taken together, these data suggested that EPHA3 regulates chemoresistance of SCLC cells through affecting the expression of PI3K/BMX/STAT3 pathway.

### EPHA3 suppresses tumor growth of SCLC cell lines in vivo

**Fig. 8:**
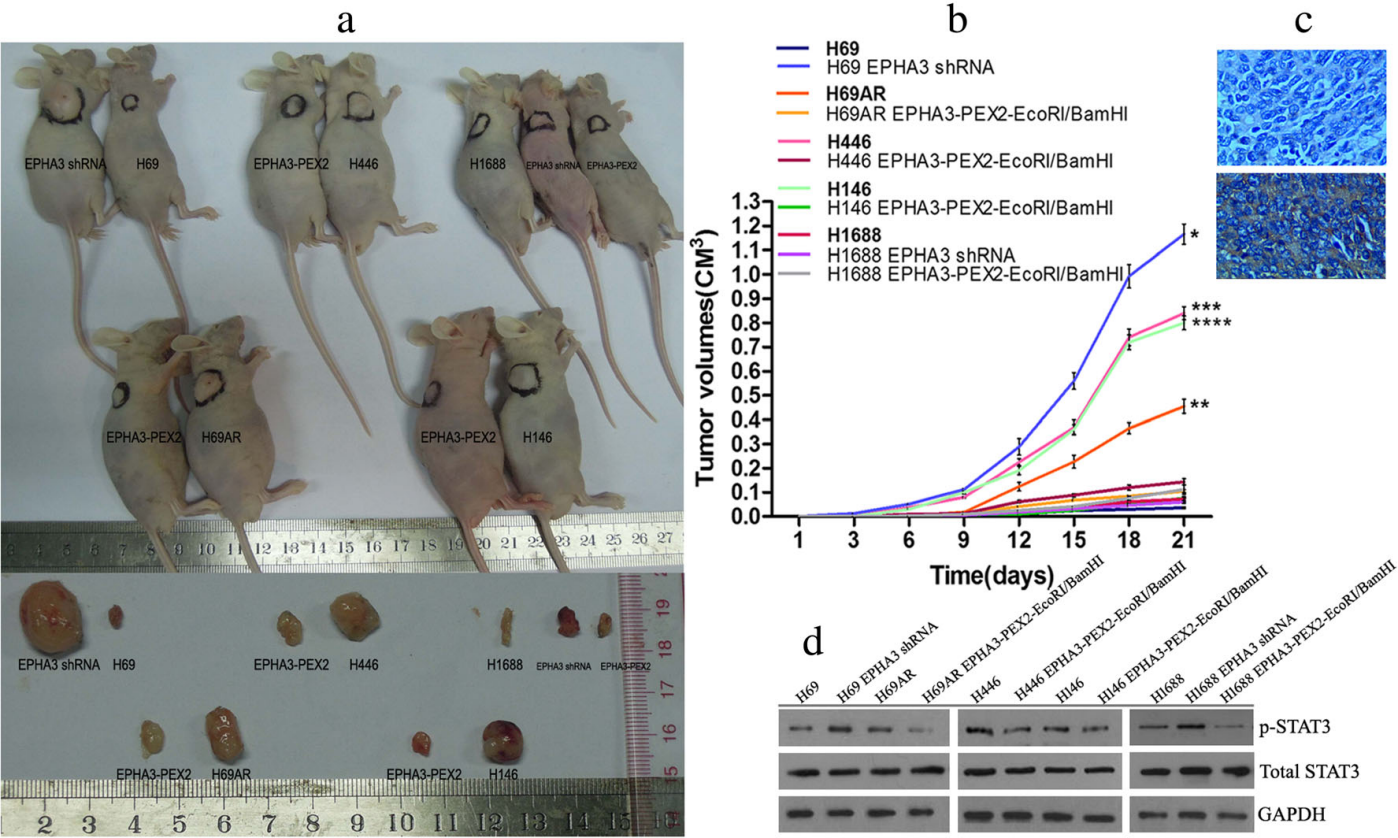
EPHA3 inhibited tumor growth of SCLC in vivo. Lower expression of EPHA3 significantly enhanced the ability of tumor growth either in H69 cells compared to H69 cells with EPHA3 deficiency or in H69AR, H446, and H146 cells compared to the corresponding cells with EPHA3 overexpression (**a**, **b**). However, a weak ability of tumor growth was shown in H1688 cells whether the expression of EPHA3 was upregulated or downregulated. The expression of EPHA3 in tumor tissues detected by immunohistochemistry (no staining and strongly positive staining, **c**) was negatively correlated with the expression level of p-STAT3 detected by Western blotting (**d**). However, no correlation was observed between the expression of EPHA3 and total STAT3 detected by Western blotting

To determine the effect of EPHA3 on tumor growth potential in vivo, SCLC cells with altered expression of EPHA3 were subcutaneously inoculated into BALB/C-nude mice. Interestingly, the results showed that lower expression of EPHA3 significantly enhanced the ability of tumor growth either in H69 cells compared to the EPHA3 downregulated cells (mean H69 tumor volumes = 36 mm^3^ vs shRNA = 1166 mm^3^, * *P* < 0.05, *t* test; Fig. 8a, b) or in H69AR, H446, and H146 cells compared to corresponding EPHA3 upregulated cells (mean H69AR tumor volumes = 455 mm^3^ vs EPHA3 = 105 mm^3^, ** *P* < 0.05, *t* test, Fig. 8a, b; mean H446 tumor volumes = 840 mm^3^ vs EPHA3 = 144 mm^3^, *** *P* < 0.05, *t* test, Fig. 8a, b; mean H146 tumor volumes = 800 mm^3^ vs EPHA3 = 75 mm^3^, **** *P* < 0.05, *t* test; Fig. 8a, b), with the exception of H1688 cells. Whether the expression of EPHA3 was upregulated or downregulated in H1688 cells, there was no significant difference in tumor growth (mean H1688 tumor volumes = 72 mm^3^ vs shRNA = 112 vs EPHA3 = 60 mm^3^, *P >* 0.05, *t* test; Fig. 8a, b). Furthermore, the expression of EPHA3 in tumors, affirmed by immunohistochemistry with 4° (the intensity of staining: high, 3; medium, 2; low, 1; no staining, 0. Fig. 8c), was observed to be negatively correlated with the expression level of p-STAT3 by Western blotting (2-tailed Spearman correlation, *r* = −0.864, *P* < 0.05; Fig. 8d; Supplementary Figure S7), but no correlation was observed with the expression level of total STAT3 (2-tailed Spearman correlation, *P* > 0.05).

### EPHA3 expression is correlated with survival time of SCLC patients

**Fig. 9:**
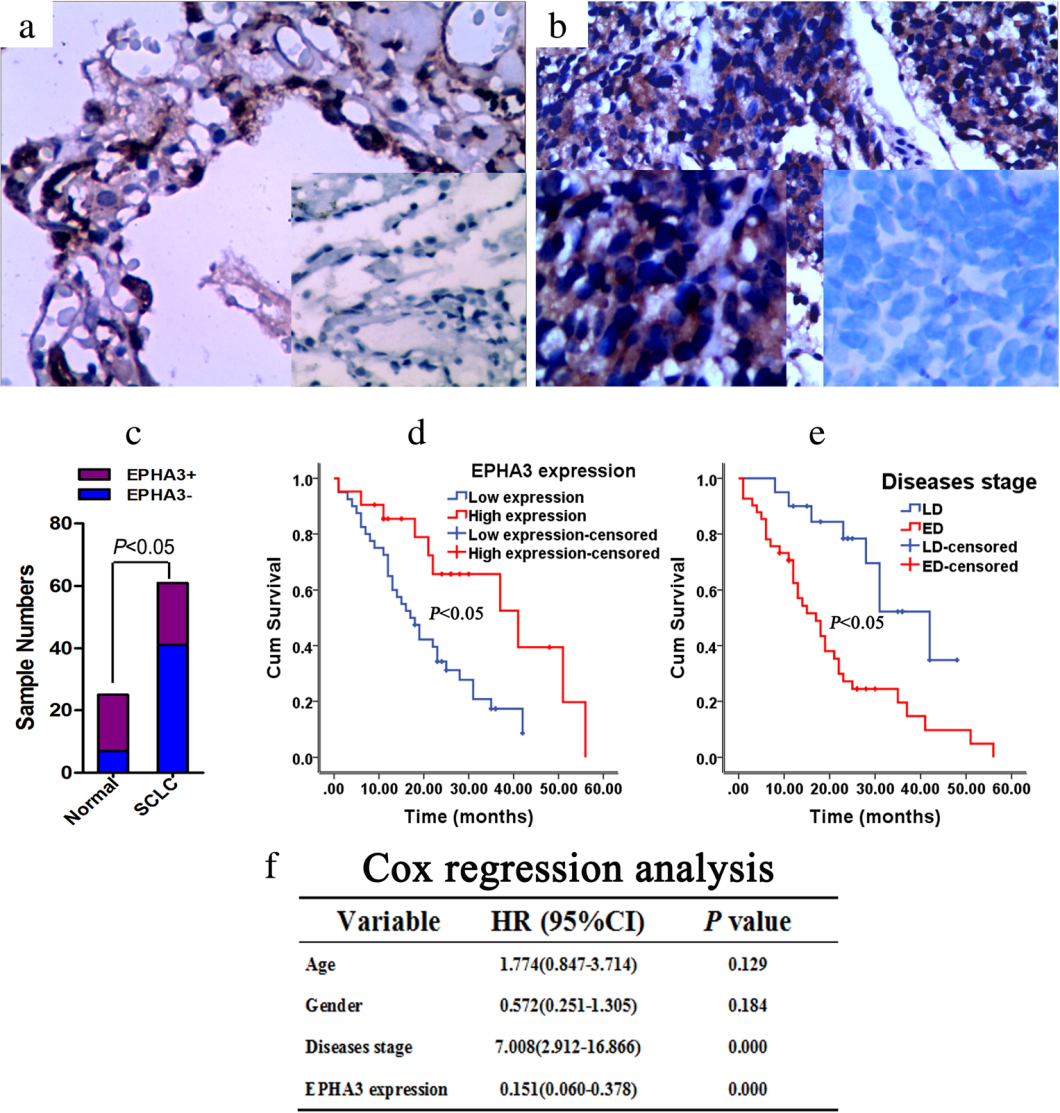
Expression of EPHA3 in diagnostic biopsy samples and the role of predicted clinical prognosis in SCLC. The expression of EPHA3 in normal lung tissue samples (72 %) (**a**) or in SCLC tumor samples (34.4 %) (**b**) was detected by immunohistochemistry (200×) with positive staining site in the cytoplasm. Significant difference was presented between them (**c**) For overall survival, the Kaplan–Meier method revealed that EPHA3 expression level (low (*blue*), high (*red*)) (**d**) and disease stage (LD (*blue*), ED (*red*)) (**e**) were correlated with significant overall survival time of the 61 SCLC patients. Cox regression analysis indicated that diseases stage and EPHA3 expression were found to be significantly independent prognostic factors for the SCLC patients (**f**)

To evaluate the clinicopathological features of EPHA3 expression in SCLC, immunohistochemical staining was performed in SCLC tissues and normal lung tissues. EPHA3 expression was detectable in 18 of 25 (72 %) normal lung tissue samples (Fig. 9a), compared with 21 of 61 (34.4 %) SCLC tumor samples (*P < 0.05, *χ* ^2^ test, Fig. 9b, c, Table 1). EPHA3 expression was correlated with the survival status of patients (**P < 0.05, *χ* ^2^ test; Table 1). However, no significant differences were observed in EPHA3 expression with respect to gender, age, and disease stage (*P >* 0.05, *χ* ^2^ test; Table 1). For overall survival, the Kaplan–Meier method revealed that EPHA3 expression level (*P* < 0.05; Fig. 9d) and disease stage (*P* < 0.05; Fig. 9e) were correlated with significant overall survival time of the 61 SCLC patients (Supplementary Table S1). Cox regression analysis indicated that disease stage and EPHA3 expression (*P* < 0.05; Fig. 9f) were found to be significantly independent prognostic factors for the SCLC patients. EPHA3 expression was an independent predictor of survival with a hazard ratio of 0.151 and a 95 % confidence interval ranging from 0.060 to 0.378.

**Table 1:** Association of EPHA3 with clinical parameters

Characteristics	EPHA3 expression		*X* ^2^	*P* value
	−	+		
SCLC cases (*N* = 61)	40	21	**10.101**	0.001*
Age			0.282	0.596
≤59	20	12		
>59	20	9		
Gender			0.091	0.763
Male	30	15		
Female	10	6		
Disease stage			2.743	0.098
Limited disease (LD)	16	4		
Extensive disease (ED)	24	17		
Survival status			**6.733**	0.009**
Survival	8	11		
Death	32	10		
Normal lung tissues (*N* = 25)	7	18		
Age			0.198	0.656
≤59	4	12		
>59	3	6		
Gender			0.103	0.748
Male	3	9		
Female	4	9		

The bold emphasis highlighted the comparison between the SCLC cases and normal cases

The symbol “*” pointed the result of *X* ^2^ test between the SCLC cases and normal cases. It has been displayed in the text “EPHA3 expression was detectable in 18 of 25 (72 %) normal lung tissue samples (Fig. 9a), compared with 21 of 61 (34.4 %) SCLC tumor samples (*P<0.05, *X* ^2^ test, Fig. 9b and c, Table 1)”

## Discussion

EphA3 was initially characterized during analysis of the retinotectal mapping of neurons [41] and was later found to be expressed in embryonic development, brain, heart, lung, bladder, prostate, and colon [8–13]. With respect to its putative role in tumorigenesis, previous studies have indicated that EPHA3 can signal both in a kinase-dependent and kinase-independent manner, inducing both tumor-promoting and tumor-suppressing effects [42, 43]. In glioblastoma multiforme, EPHA3 has exhibited highly expression in undifferentiated mesenchymal cells and has been especially assigned a kinase-independent oncogenic role based on its modulating mitogen-activated protein kinase (MAPK) signaling [15]. However, in a series of studies of non-small cell lung cancer (NSCLC), EPHA3 was identified as a tumor suppressor with decreased expression levels. The reduction in receptor activity conferred by the point mutations of EPHA3 found in cancers, and ligand- and EPHA3-dependent apoptosis of tumor and stroma cells upon receptor agonist treatment suggested that wild-type EPHA3 has anti-tumorigenic properties in NSCLC [19, 43–46].

Given the opposing outcomes of aberrant EPHA3 expression in different tumors, as well as our previous results that the expression of EPHA3 in H69AR was lower than in the parent cell line H69, we proposed a hypothesis that EPHA3 might be involved in regulating the MDR of SCLC. In this study, we manipulated the expression of EPHA3 through loss- and gain-of-function approaches in 5 SCLC cell lines to explore the drug resistance mediated by EPHA3. Both approaches clearly indicated that EPHA3 improves chemosensitivity and suppresses tumor growth in vivo, which manifests that EPHA3 acts as a tumor suppressor in SCLC. Furthermore, higher expression levels of EPHA3 in SCLC tumor samples were correlated with longer overall survival of patients, implying that EPHA3 might be associated with chemosensitivity. Overall, our findings are consistent with the results from NSCLC [19] and confirm that EPHA3 is involved in regulating the chemoresistance of SCLC with tumor-suppressing effects.

Considering the observation that re-expression of wild-type EPHA3 in H1299 cells increased apoptosis by suppression of AKT activation in vitro [19], as well as that the AKT/mTOR pathway was upregulated in SCLC, and inhibiting mTOR signaling with RAD001 potently disrupted growth and survival signaling in human SCLC cells [47], we proposed the hypothesis that the upstream gene of AKT, PI3K, may be involved in apoptosis of SCLC cells. Recent studies show that PIP3 (the product of PI3K) serves as the branching point for two pathways, the PI3K/AKT/TOR signaling pathway and the PI3K/BMX/STAT3 signaling pathway, that both contribute to the oncogenic cellular phenotype. The former has been studied in great deal, but the latter has not yet been identified [20]. To gain insight into the molecular basis of the role of EPHA3 in modulating MDR potentially through the PI3K/BMX/STAT3 signaling pathway, we detected the protein expression of PI3K/BMX/STAT3 in these SCLC cell lines. Our results suggested that upregulation of EPHA3 inhibits the phosphorylation of PI3K/BMX/STAT3 signaling pathway, especially p85α expression, which is consistent with the observation that the nSH2 domain of p85 specifically interacts with the kinase domain of EPH receptor [29]. Furthermore, the expression pattern of p-STAT3 was negatively correlated with cell apoptosis induced by chemotherapeutic drugs, which confirmed that EPHA3 could influence cell apoptosis through PI3K/BMX/STAT3 signaling to modulate MDR in SCLC cells. Moreover, both PI3K inhibitor LY294002 and BMX inhibitor LFM-A13 reduced the phosphorylation of BMX and/or STAT3 in the stably EPHA3 silenced SCLC cells, and increased sensitivity of these cells to chemotherapy drugs, which coincided with the observation that the survival of the cisplatin-resistant SCLC cell lines was well suppressed by BEZ235, accompanied by the suppression of S6RP phosphorylation [48]. It was noteworthy that the expression of EPHA3 in tumors was observed to be negatively correlated with the expression level of p-STAT3, which was consistent with the results in the cell lines. These results provided more evidences to show that EPHA3 stimulates apoptosis via PI3K/BMX/STAT3 signaling. However, our data failed to explain how EPHA3 acts on PI3K through p85α in SCLC, which need further research to clarify.

Considerable research has indicated that the PI3K/AKT/mTOR pathway plays an important role on chemoresistance of SCLC. Genetic alterations in the PI3K/AKT/mTOR pathway were detected in 36 % of the 51 SCLC tumor samples [48]. The AKT/mTOR pathway was upregulated in SCLC and RAD001 sensitized human SCLC cells to etoposide [47, 49]. A recent report investigated the antitumor effects of three mTOR inhibitors including everolimus in 7 SCLC cell lines and revealed that only SBC5 cells showed sensitivity to everolimus. Furthermore, eIF4E was shown to be an important factor in the resistance to everolimus in SCLC cells, and a link between MYC and mTOR-independent eIF4E contributed to the resistance to everolimus in SCLC cells [50]. In view of these results, we need extendable studies to verify whether EPHA3 has an impact on downstream AKT/mTOR signaling.

In summary, our study revealed a coordinated downregulation of EPHA3 gene expression in SCLC multidrug-resistance cells, whereas upregulation of EPHA3 impaired chemoresistance by inducing cell apoptosis via the PI3K/BMX/STAT3 signaling pathway. This study provides a novel insight into the mechanism of chemoresistance mediated by EPHA3. Our data suggests that EPHA3 plays a tumor suppressor role and may be a candidate target for developing therapeutic strategies to overcome drug resistance in SCLC.

## Electronic supplementary material

Below is the link to the electronic supplementary material.

Supplementary Figure S1 Over-expression of EPHA3 induced the cell early apoptosis rate and G0/G1 phase arrest as illustrated by the representative FACS profiles. H69AR (A&D), H446 (B&E) and H146 (C&F) cells transfected with plasmid EPHA3-PEX2-EcoRI/BamHI or NC were detected by flow cytometric analysis after treated with ADM, DDP and VP-16. (GIF 529 kb)

High resolution image (TIF 44903 kb)

Supplementary Figure S2 SCLC cell lines transfected with EPHA3 shRNA. The EPHA3 shRNA-1690 for H69 and H69AR cells, as well as −2934 for H446, H146 and H1688 cells were transfected into the cell lines. (GIF 39388 kb)

High resolution image (TIF 126595 kb)

Supplementary Figure S3 Representative FACS profiles showed that knockdown of EPHA3 resulted in a reduced early apoptosis rate and G2/M cell-cycle arrest. Cell apoptosis and cell-cycle were assayed by flow cytometric analysis after H69 (A&C) and H1688 (B&D) cells with EPHA3 deficiency were treated with ADM, DDP and VP-16. (GIF 377 kb)

High resolution image (TIF 39758 kb)

Supplementary Figure S4 Re-expression of EPHA3 increased the cell early apoptosis rate. Cell apoptosis were assayed by flow cytometric analysis after H69 (A) and H1688 (B) cells co-transfection with plasmid EPHA3-PEX2-EcoRI/BamHI were treated with ADM, DDP and VP-16. (GIF 5088 kb)

High resolution image (TIF 28570 kb)

Supplementary Figure S5 Column bar graphs for the expression of PI3K/BMX/STAT3 signaling protein. The protein expression of p-PI3K-p85α (A), p-BMX (B), p-STAT3 (C), total PI3K-p85α (D), total BMX (E) and total STAT3 (F) was modulated by up- or down-regulation of EPHA3 in SCLC cell lines. (GIF 139 kb)

High resolution image (TIF 1470 kb)

Supplementary Figure S6 The expression of phosphorylated signaling proteins was blocked by the signaling pathway inhibitors. The protein expression of p-PI3K-p85α (A), p-BMX (B), p-STAT3 (C), total PI3K-p85α (D), total BMX (E) and total STAT3 (F) in the stably silenced cells was regulated by the inhibition of PI3K/BMX pathway with LY294002 or the inhibition of BMX/STAT3 pathway with LFM-A13. (GIF 98 kb)

High resolution image (TIF 1222 kb)

Supplementary Figure S7 The relative expression of EPHA3, p-STAT3 and total STAT3 in tumors of mice. The expression of EPHA3 in tumor tissues detected by immunohistochemistry was negatively correlated with the expression level of p-STAT3 detected by Western blotting, but shown no correlation with the expression level of total STAT3. (GIF 41 kb)

High resolution image (TIF 5618 kb)

Supplementary Table 1 (DOC 41 kb)
