# Supplementary figures and images for: EPHA3 regulates the multidrug resistance of small cell lung cancer via the PI3K/BMX/STAT3 signaling pathway
Source: Tumour Biol. 2016 Apr 21;37(9):11959–71. doi: 10.1007/s13277-016-5048-4 (PMC5080350; doi:10.1007/s13277-016-5048-4)

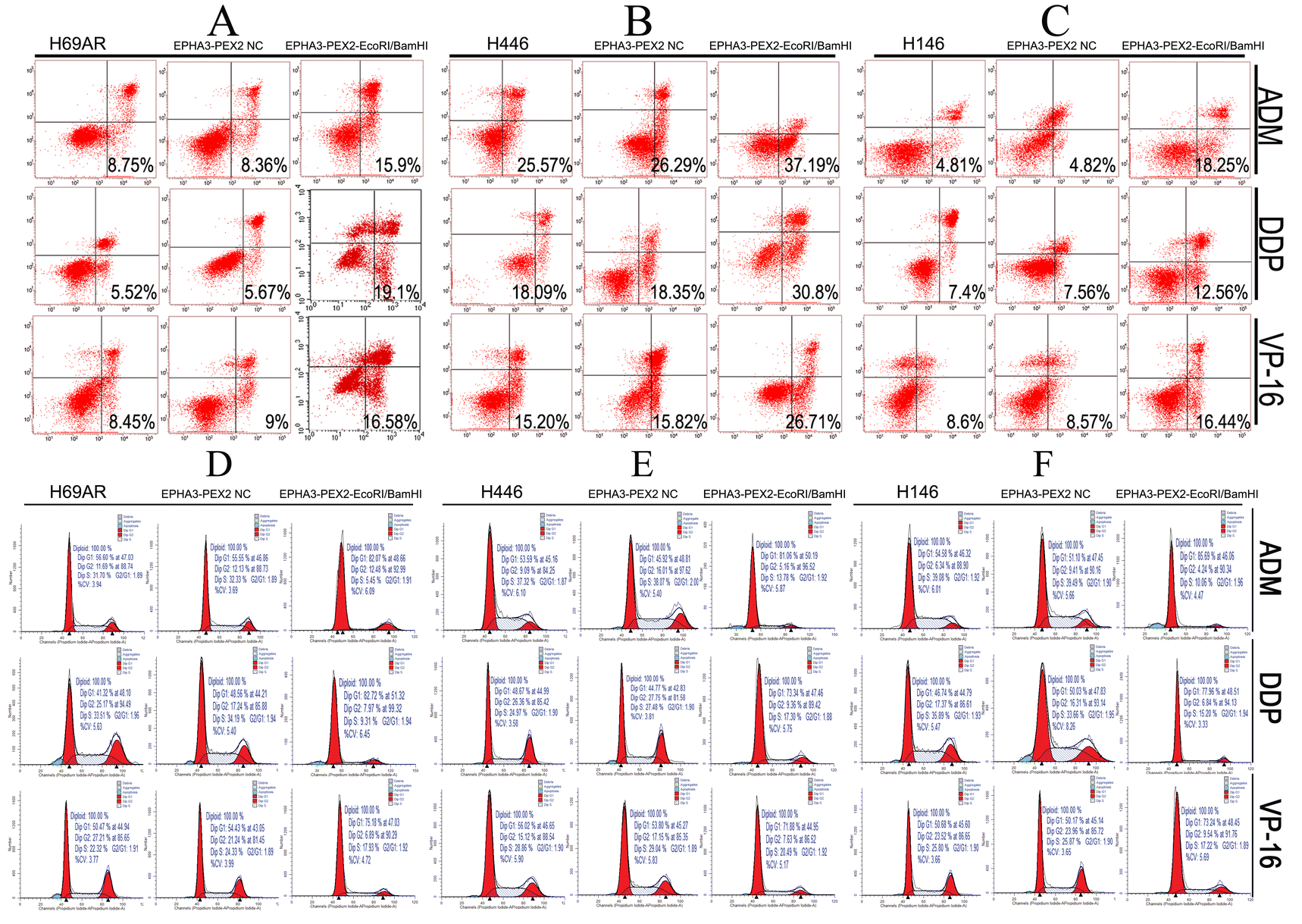

Supplement: Supplementary file 1 — Over-expression of EPHA3 induced the cell early apoptosis rate and G0/G1 phase arrest as illustrated by the representative FACS profiles. H69AR (A&D), H446 (B&E) and H146 (C&F) cells transfected with plasmid EPHA3-PEX2-EcoRI/BamHI or NC were detected by flow cytometric analysis after treated with ADM, DDP and VP-16. (GIF 529 kb) [file 13277_2016_5048_Fig10_ESM.gif]

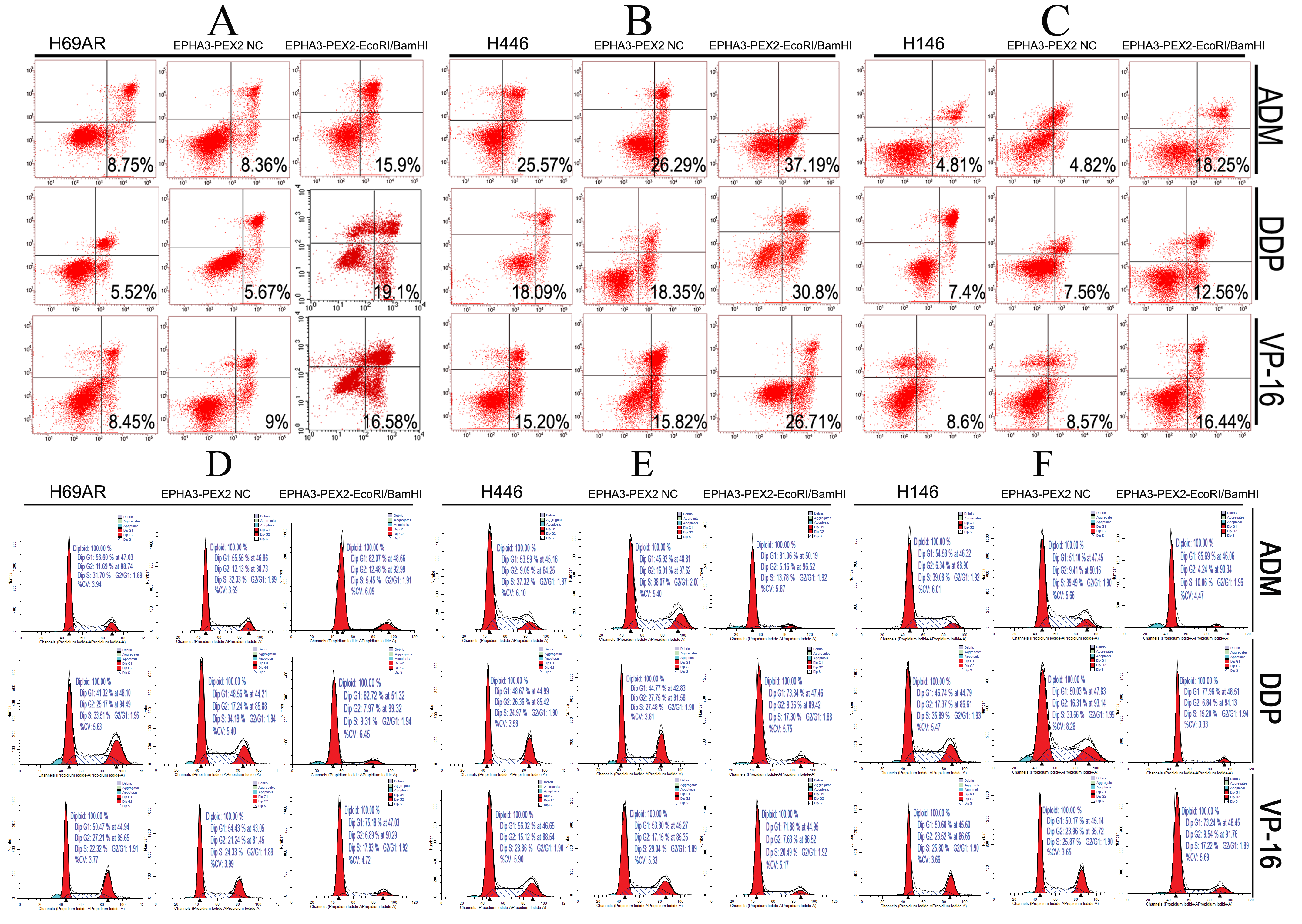

Supplement: Supplementary file 2 — High resolution image (TIF 44903 kb) [file 13277_2016_5048_MOESM1_ESM.tif]

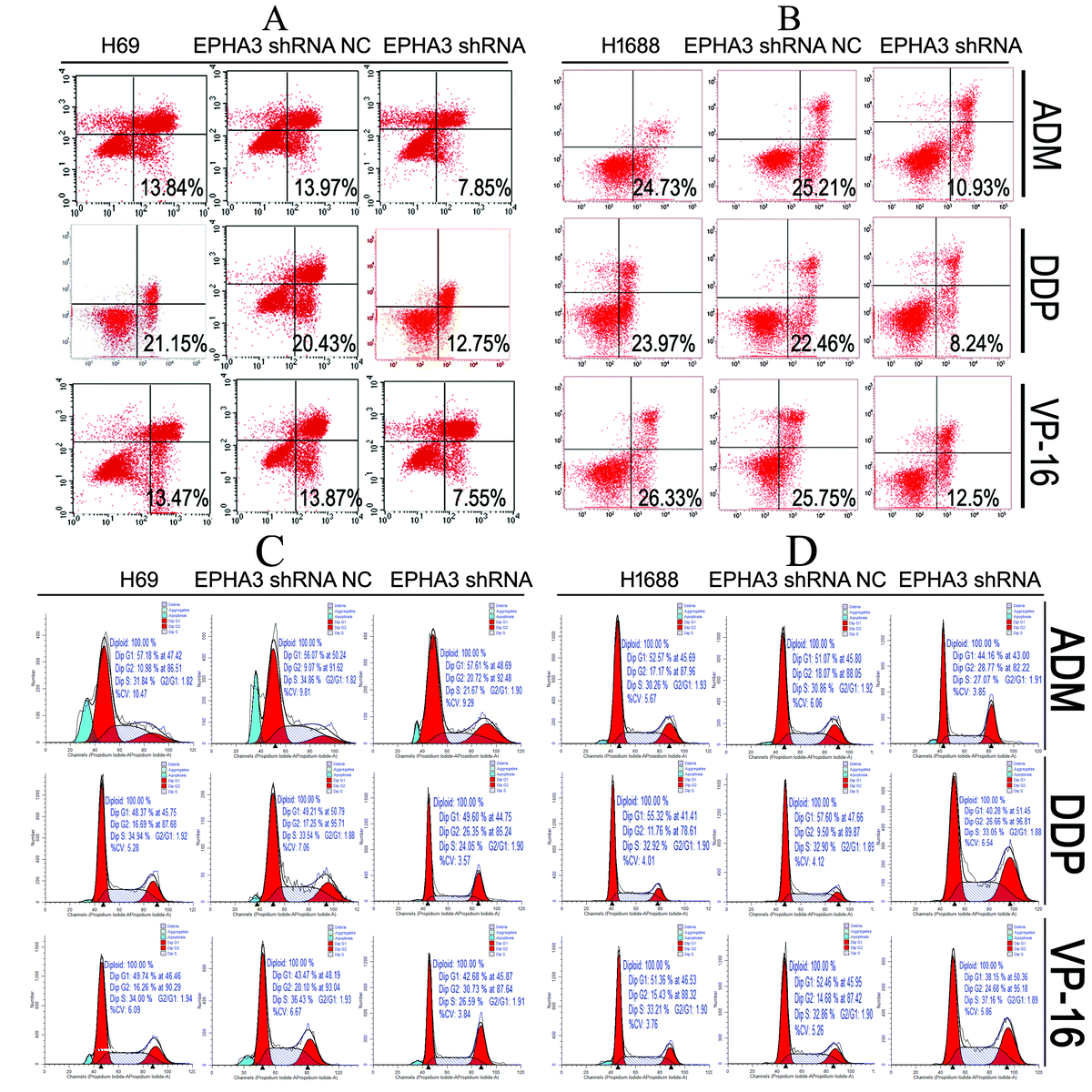

Supplement: Supplementary file 5 — Representative FACS profiles showed that knockdown of EPHA3 resulted in a reduced early apoptosis rate and G2/M cell-cycle arrest. Cell apoptosis and cell-cycle were assayed by flow cytometric analysis after H69 (A&C) and H1688 (B&D) cells with EPHA3 deficiency were treated with ADM, DDP and VP-16. (GIF 377 kb) [file 13277_2016_5048_Fig12_ESM.gif]

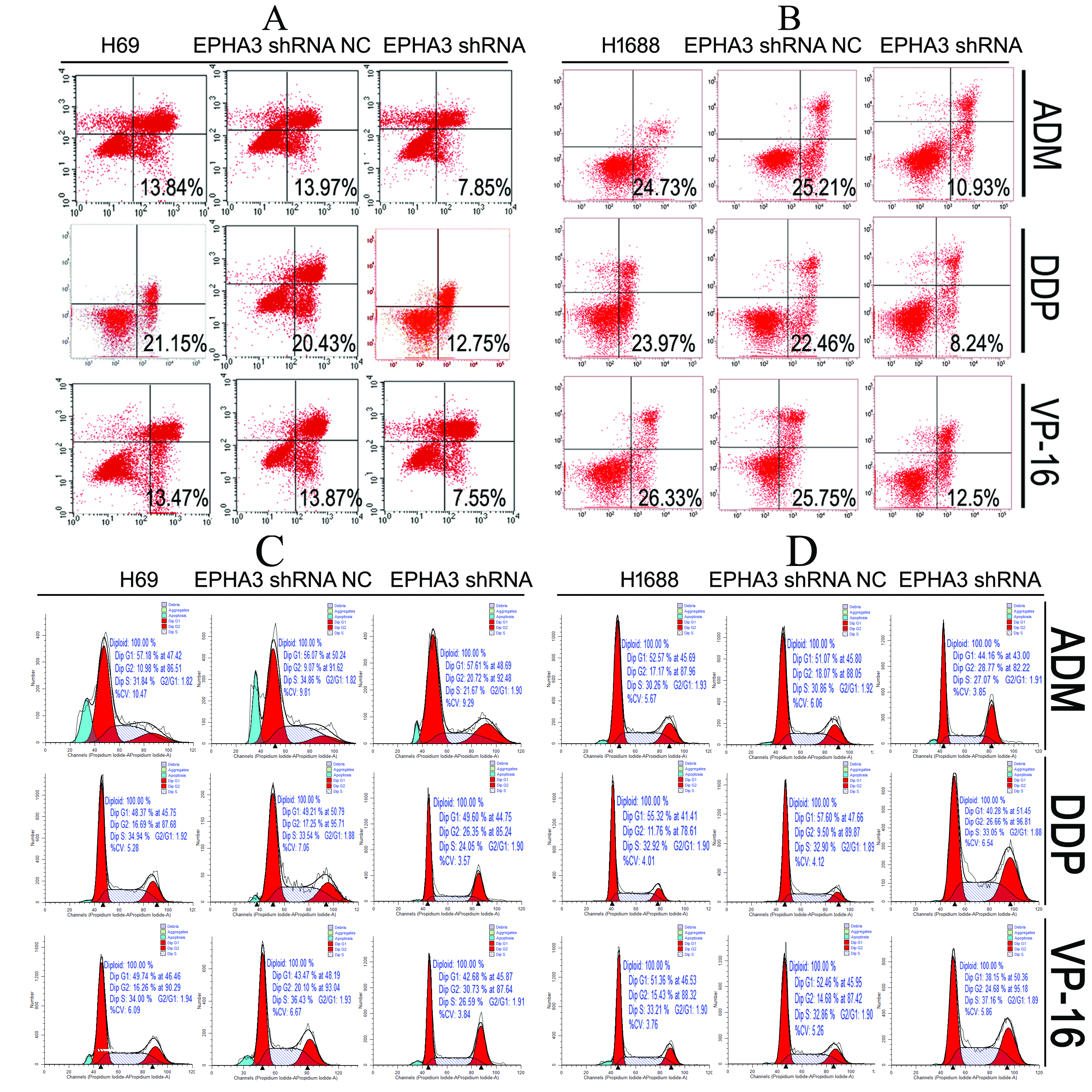

Supplement: Supplementary file 6 — High resolution image (TIF 39758 kb) [file 13277_2016_5048_MOESM3_ESM.tif]

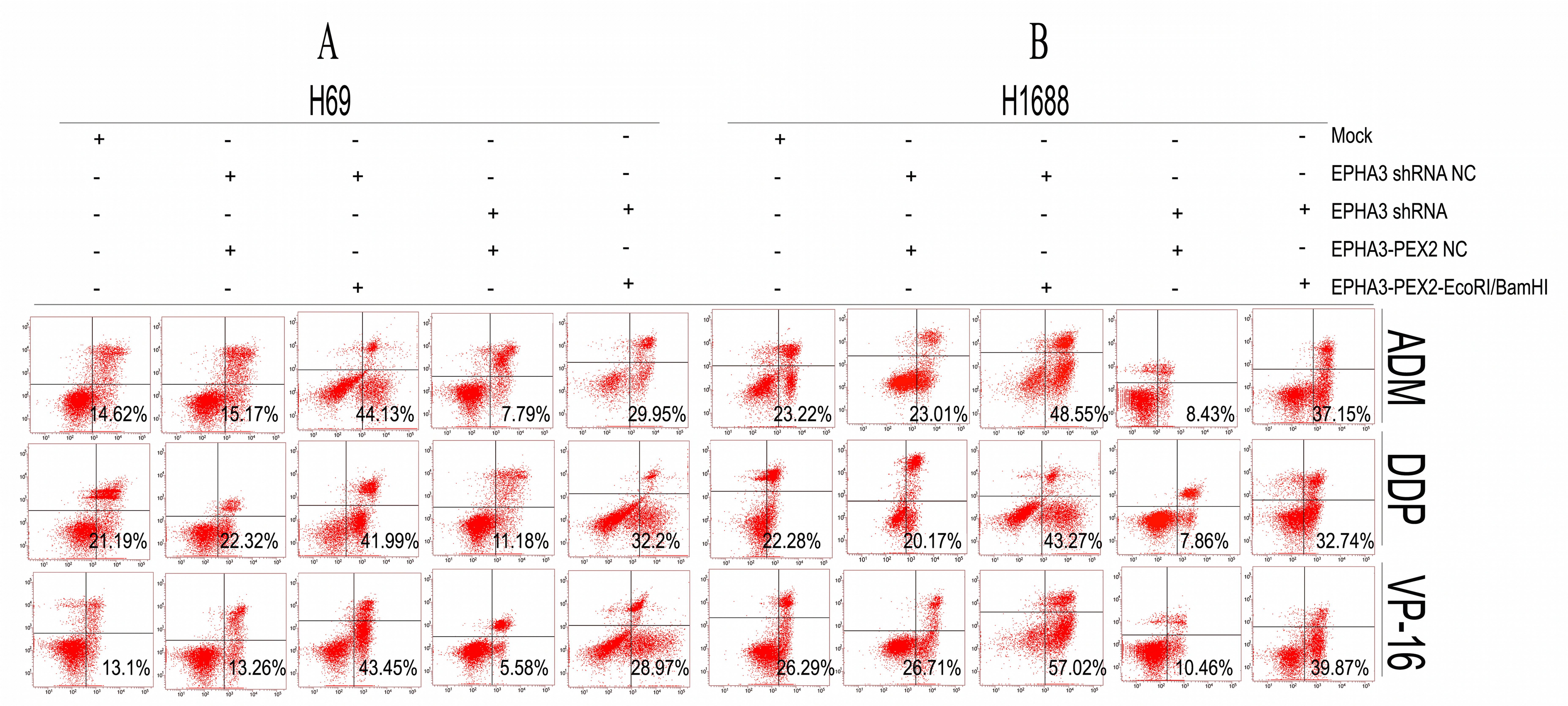

Supplement: Supplementary file 7 — Re-expression of EPHA3 increased the cell early apoptosis rate. Cell apoptosis were assayed by flow cytometric analysis after H69 (A) and H1688 (B) cells co-transfection with plasmid EPHA3-PEX2-EcoRI/BamHI were treated with ADM, DDP and VP-16. (GIF 5088 kb) [file 13277_2016_5048_Fig13_ESM.gif]

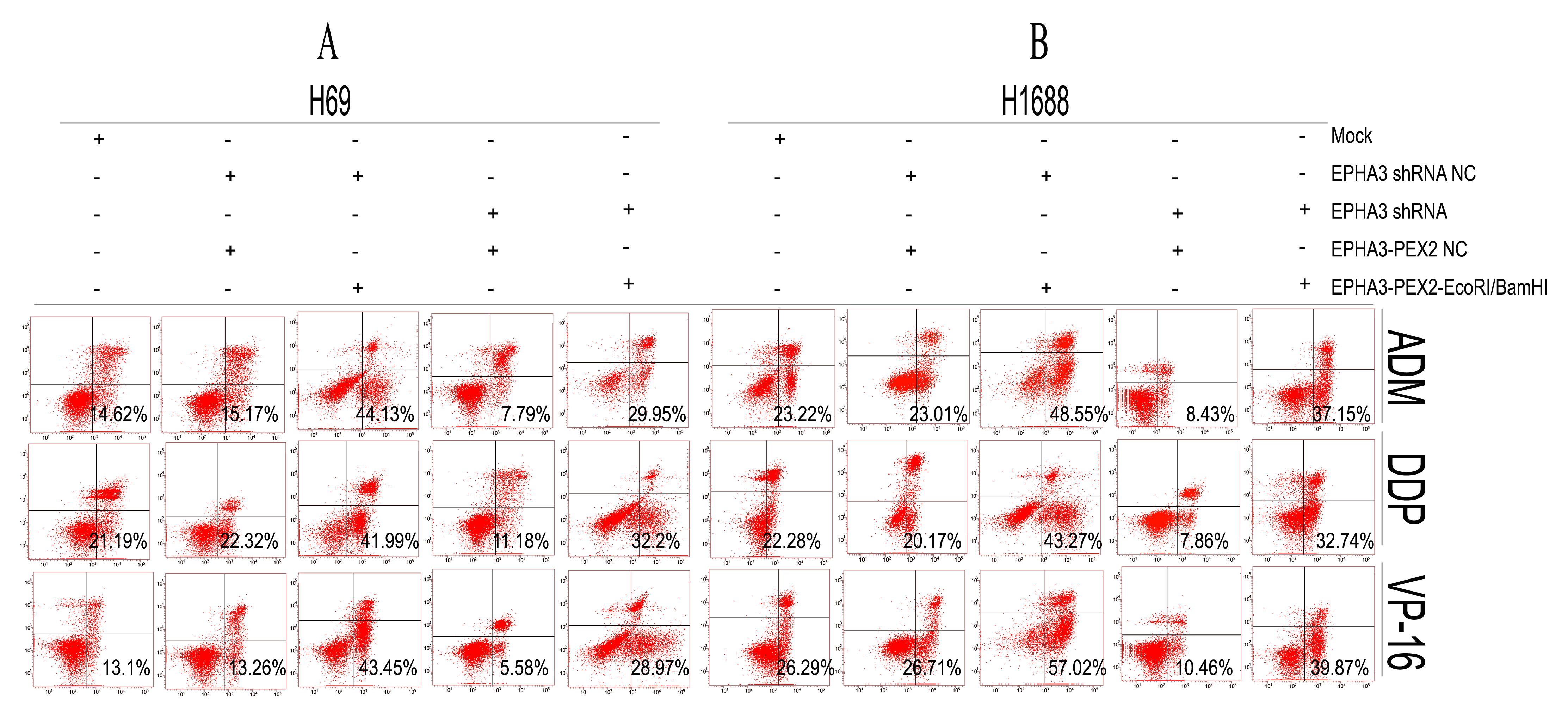

Supplement: Supplementary file 8 — High resolution image (TIF 28570 kb) [file 13277_2016_5048_MOESM4_ESM.tif]

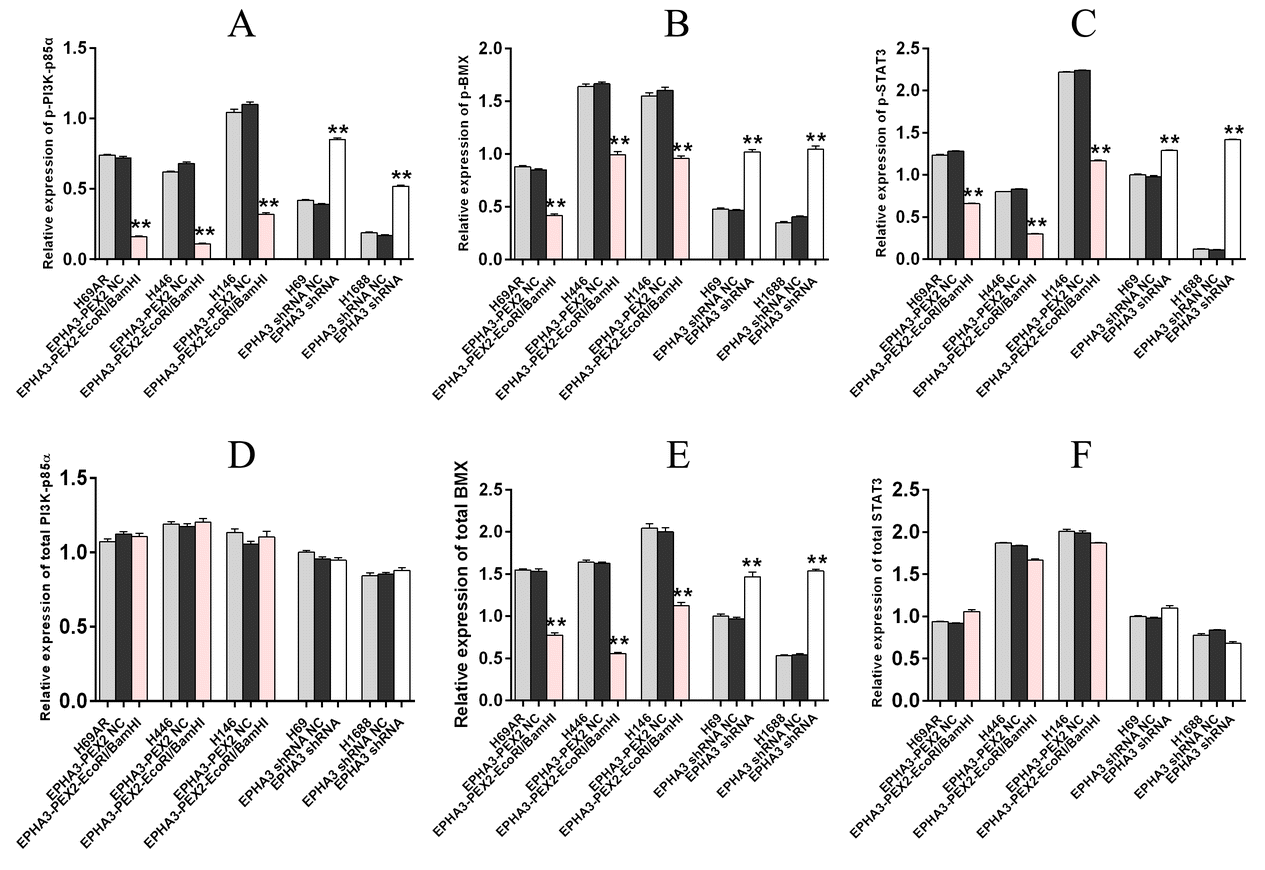

Supplement: Supplementary file 9 — Column bar graphs for the expression of PI3K/BMX/STAT3 signaling protein. The protein expression of p-PI3K-p85α (A), p-BMX (B), p-STAT3 (C), total PI3K-p85α (D), total BMX (E) and total STAT3 (F) was modulated by up- or down-regulation of EPHA3 in SCLC cell lines. (GIF 139 kb) [file 13277_2016_5048_Fig14_ESM.gif]

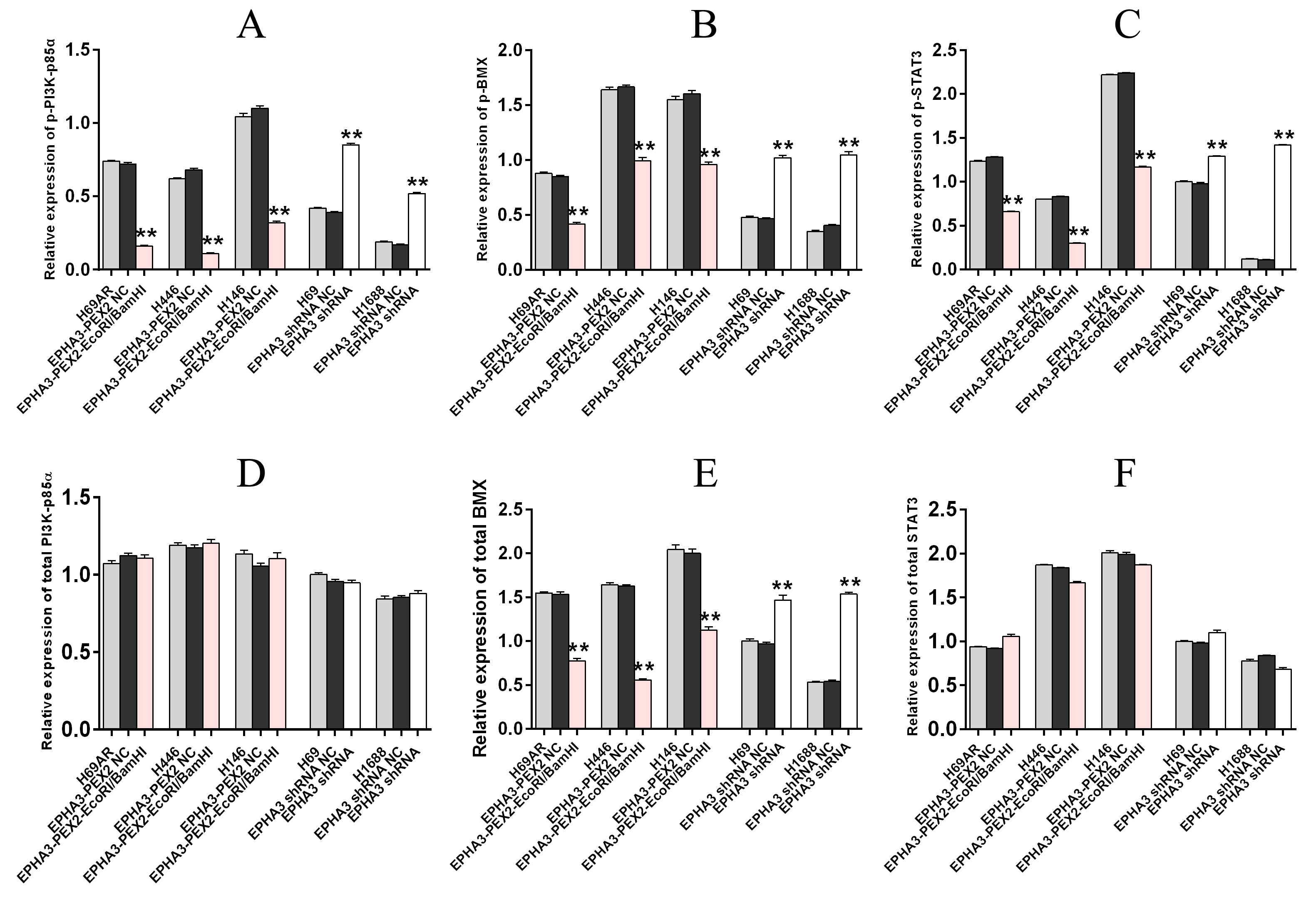

Supplement: Supplementary file 10 — High resolution image (TIF 1470 kb) [file 13277_2016_5048_MOESM5_ESM.tif]

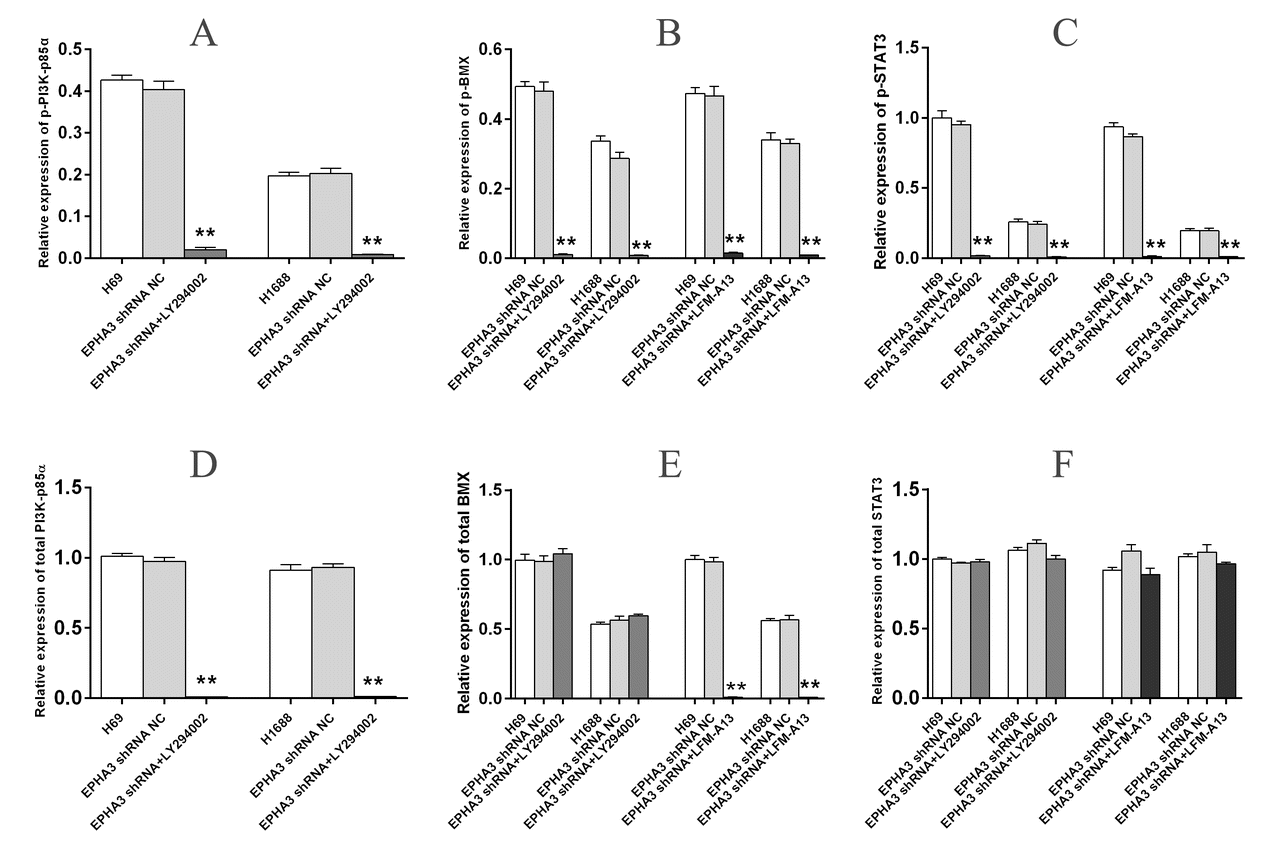

Supplement: Supplementary file 11 — The expression of phosphorylated signaling proteins was blocked by the signaling pathway inhibitors. The protein expression of p-PI3K-p85α (A), p-BMX (B), p-STAT3 (C), total PI3K-p85α (D), total BMX (E) and total STAT3 (F) in the stably silenced cells was regulated by the inhibition of PI3K/BMX pathway with LY294002 or the inhibition of BMX/STAT3 pathway with LFM-A13. (GIF 98 kb) [file 13277_2016_5048_Fig15_ESM.gif]

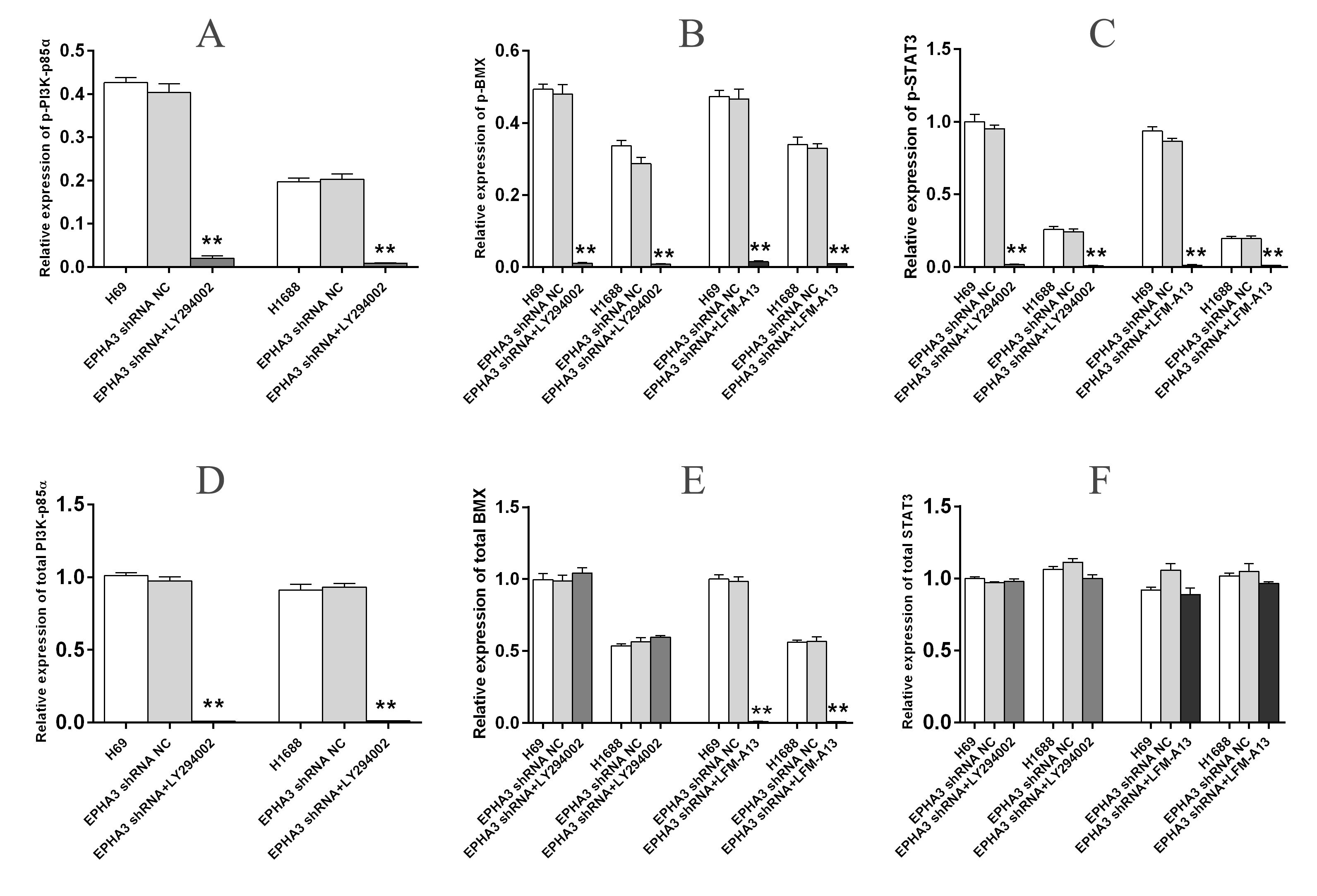

Supplement: Supplementary file 12 — High resolution image (TIF 1222 kb) [file 13277_2016_5048_MOESM6_ESM.tif]

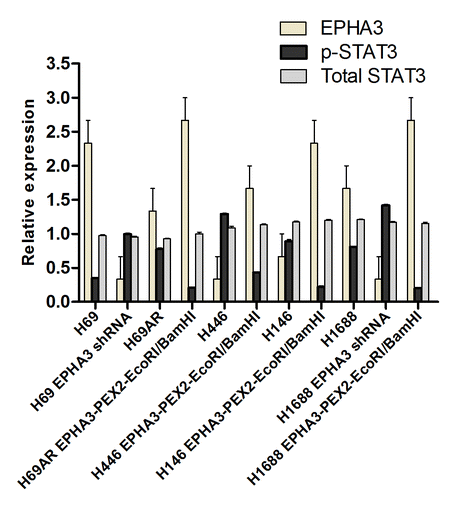

Supplement: Supplementary file 13 — The relative expression of EPHA3, p-STAT3 and total STAT3 in tumors of mice. The expression of EPHA3 in tumor tissues detected by immunohistochemistry was negatively correlated with the expression level of p-STAT3 detected by Western blotting, but shown no correlation with the expression level of total STAT3. (GIF 41 kb) [file 13277_2016_5048_Fig16_ESM.gif]

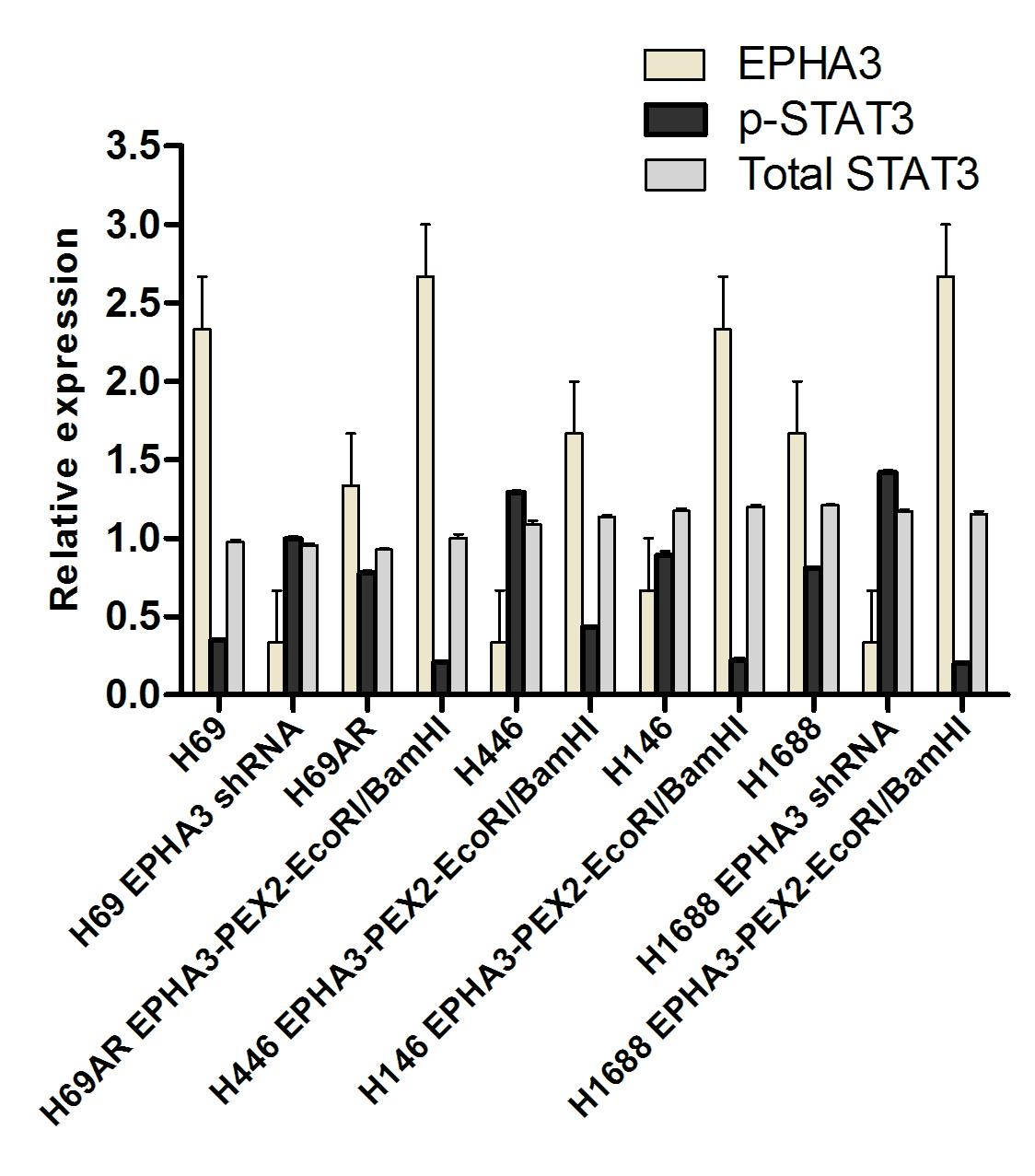

Supplement: Supplementary file 14 — High resolution image (TIF 5618 kb) [file 13277_2016_5048_MOESM7_ESM.tif]
