# Supplementary material for: EPHA3 regulates the multidrug resistance of small cell lung cancer via the PI3K/BMX/STAT3 signaling pathway
Source: Tumour Biol. 2016 Apr 21;37(9):11959–71. doi: 10.1007/s13277-016-5048-4 (PMC5080350; doi:10.1007/s13277-016-5048-4)
Supplement: Supplementary file 15 — (DOC 41 kb) [file 13277_2016_5048_MOESM8_ESM.doc]

Supplementary Table S1. The Kaplan-Meier analysis of relations between survival times and EPHA3 expression in 61 cases of small cell lung cancer patients with clinical pathologic characteristics

| Patients characteristics | | N | Mean survival time(months) | Median survival time(months) | X^2^ | *P value* |
| --- | --- | --- | --- | --- | --- | --- |
| Gender | |  |  |  | 0.227 | 0.634 |
|  | Male | 45 | 26.163 | 23 |  |  |
|  | Female | 16 | 23.583 | 21 |  |  |
| Age | |  |  |  | 2.841 | 0.092 |
|  | ≤59 | 32 | 21.162 | 19 |  |  |
|  | ＞59 | 29 | 29.949 | 25 |  |  |
| Disease stage | |  |  |  | **10.265** | **0.001** |
|  | Limited disease (LD) | 20 | 35.098 | 42 |  |  |
|  | Extensive-stage disease (ED) | 41 | 20.249 | 17 |  |  |
| EPHA3 expression | |  |  |  | **6.997** | **0.008** |
|  | - | 40 | 20.260 | 17 |  |  |
|  | + | 21 | 36.249 | 41 |  |  |
